# Recent Advances in Asymmetric Catalysis Using p‐Block Elements

**DOI:** 10.1002/anie.202316461

**Published:** 2023-12-18

**Authors:** Milan Pramanik, Michael G. Guerzoni, Emma Richards, Rebecca L. Melen

**Affiliations:** ^1^ Cardiff Catalysis Institute School of Chemistry Cardiff University Translational Research Hub Maindy Road Cathays, Cardiff CF24 4HQ Cymru/Wales UK

**Keywords:** Aluminium, Bismuth, Boron, Enantioselective Catalysis, Frustrated Lewis Pairs

## Abstract

The development of new methods for enantioselective reactions that generate stereogenic centres within molecules are a cornerstone of organic synthesis. Typically, metal catalysts bearing chiral ligands as well as chiral organocatalysts have been employed for the enantioselective synthesis of organic compounds. In this review, we highlight the recent advances in main group catalysis for enantioselective reactions using the p‐block elements (boron, aluminium, phosphorus, bismuth) as a complementary and sustainable approach to generate chiral molecules. Several of these catalysts benefit in terms of high abundance, low toxicity, high selectivity, and excellent reactivity. This minireview summarises the utilisation of chiral p‐block element catalysts for asymmetric reactions to generate value‐added compounds.

## Graphical abstract

The development of new methods for enantioselective reactions that generate stereogenic centres within molecules is a cornerstone of organic synthesis. In this minireview we highlight the recent advances in enantioselective main group catalysis of the p‐block elements including boron, phosphorus, bismuth and aluminium.

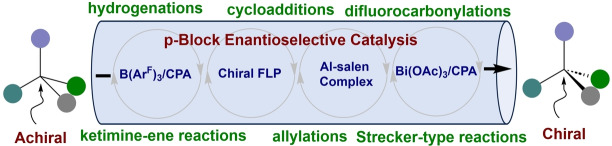

## 1 Introduction

Breaking symmetry to build molecular asymmetricity is a highly appealing synthetic approach for designing complex chiral architectures. Inspired by nature, the synthetic chemist has emulated different approaches for the synthesis of chiral molecules. In this context, asymmetric catalysis is one of the most important and prominent synthetic approaches that has significantly expanded the asymmetric toolbox in organic synthesis.[^1^, ^2^] In particular, asymmetric transition metal catalysis using chiral ligands has dominated this field and has solved many stereospecific challenges due to the flexible oxidation states and accessibility of the d‐orbitals.^[3]^ In spite of the several advantages of using transition metal catalysts, this strategy has some drawbacks owing to the high cost, low abundance, and toxicity issues of several metals. In particular, in the pharmaceutical sector, this is problematic due to isolation and separation problems of the final drug molecule from toxic metal impurities. Decades later, asymmetric organo‐catalysis significantly boosted this research field providing alternatives to the use of transition metals.[^4^, ^5^, ^6^] Indeed, this field has become prevalent, and pioneers in the field List and MacMillan were awarded the Nobel prize in 2021.^[7]^

Recently, related efforts in main group catalysis, using other elements in the s‐ and p‐block of the periodic table, have garnered interest as alternatives to organocatalysts for the transition metal free construction of enantioenriched skeletons. In particular, several s‐block elements offer a cheap and non‐toxic alternative to transition metals. For example, electropositive alkaline earth metal complexes such as magnesium, calcium, and strontium when chelated with chiral ligands have been shown to induce chirality in various organic transformations. Many timely reviews have also appeared on this topic.[^8^, ^9^] Among the group 13 elements, boron has also found widespread use in asymmetric catalysis due to its adjustable Lewis acidity, and formation of boron‐centred adducts.[^10^, ^11^, ^12^] Boron‐containing molecules can be easily embedded in chiral ligands, usually through the hydroboration of unsaturated bonds, to afford chiral Lewis acids, which can then promote highly enantioselective reactions such as the hydrogenation of imines,[^13^, ^14^, ^15^] Diels–Alder reactions,[^16^, ^17^, ^18^] and intramolecular hydroalkoxylation reactions.^[19]^ In addition, the expanding field of Frustrated Lewis Pair (FLP) chemistry originating from the combination of sterically encumbered chiral Lewis acidic boranes and achiral Lewis bases or *vice versa* has advanced the chemical landscape of main group enantioselective transformations.[^20^, ^21^, ^22^, ^23^] Moving down group 13, aluminium has often been employed in enantioselective catalysis using chiral ligands.^[24]^ Commonly, the aluminium centre is embedded in salen complexes to promote reactions such as the enantioselective hydroborations of ketones,^[25]^ carboxycyanations of aldehydes,[^26^, ^27^] desymmetrisation of meso‐epoxides,^[28]^ and hydrocyanations of aldimines.^[29]^ Despite these successful reports, Al‐salen complexes suffer some drawbacks in their wider application in asymmetric catalysis due to their hydrolytic instability, sensitivity towards moisture and light, and tendency to undergo ligand exchange processes.^[30]^ When considering the heavier group 15 elements, bismuth has gained popularity in catalysis in recent years,^[31]^ and has also been found to show applications in enantioselective synthesis. For example, Bi(OAc)_3_/chiral phosphoric acid (CPA) systems have been utilised for enantioselective allylation reactions,[^32^, ^33^, ^34^, ^35^, ^36^] difluorocarbonylations,^[37]^ and aza‐Friedel–Crafts reactions.^[38]^ In this mini‐review we will highlight the key discoveries made in recent years (since 2016) in enantioselective catalysis employing borane, FLPs, bismuth, and aluminium as p‐block catalysts.

## 2 Enantioselective Reactions using p‐Block Elements

### 2.1 Borane Catalysis using Cooperative Chiral Ligands or Chiral Boranes

**Figure 1:**
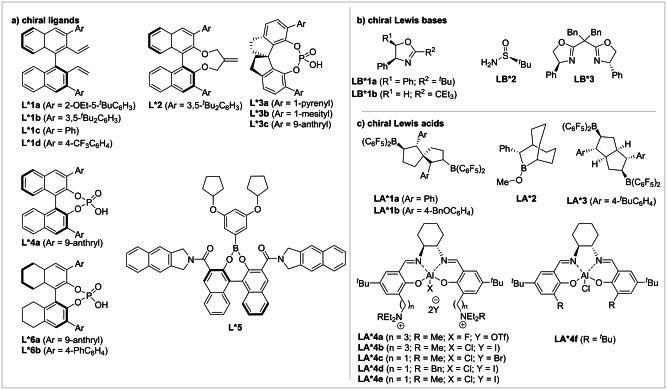
List of a) chiral ligands, b) chiral Lewis bases, and c) chiral Lewis acids discussed in this review.

Chiral boranes are well established in the literature and have commonly been employed under both stoichiometric and catalytic conditions.^[11]^ A common strategy used in recent developments of chiral borane catalysts is to synthesise the chiral borane in situ from the hydroboration reaction of chiral substituted alkenes or alkynes. Recently, this strategy has been pioneered by Du and co‐workers in the hydroboration of a chiral diene or diyne with the Lewis acidic borane HB(C_6_F_5_)_2_ (Piers’ borane).[^39^, ^40^, ^41^, ^42^, ^43^, ^44^, ^45^] One of the familiar methods to exert asymmetric induction relies on the use of a 1,1′‐bi‐2‐naphthol (BINOL)‐derived ligand tethered with two unsaturated bonds as in ligand **L1** (Figure 1).

**Scheme 1:**
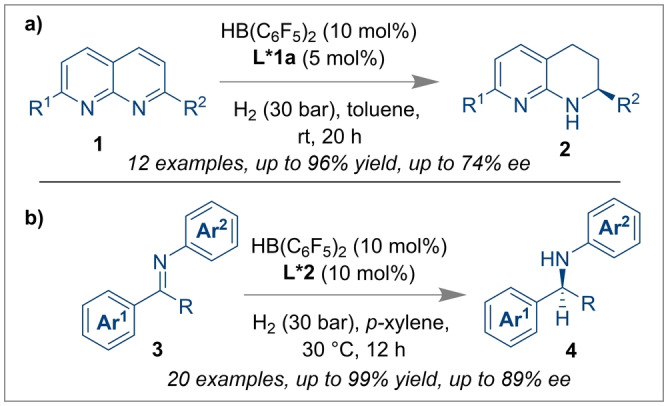
Enantioselective hydrogenation of N‐containing substrates using Piers’ borane and a BINOL‐based chiral ligand.

For example, Du et al. found that the hydroboration product between the chiral diene *(S)‐* L*1 a and HB(C_6_F_5_)_2_ promotes the hydrogenation of 2,7‐disubstituted 1,8‐naphthyridines **1** to afford a variety of 1,2,3,4‐tetrahydro‐1,8‐naphthyridines **2** in high yields and enantioselectivities (96 % yield and 74 % *ee*) (Scheme 1a).^[46]^ While investigating the regioselectivity of the reaction, the authors realised that hydrogenation could selectively be attained for 1,8 naphthyridine derivatives having R^1^=aryl and R^2^=alkyl. In a subsequent study, the hydrogenation reaction was generalised to imines **3** and the stereoselectivity was improved by using the chiral alkene *(R)‐* L*2 in the presence of 10 mol % Piers’ borane.^[13]^ In this case, the chiral ligand was also based upon a BINOL backbone but only contained one alkene site for hydroboration generating a chiral mono‐borane in situ as opposed to the proposed bis‐borane catalyst that is formed in situ for *(S)‐* L*1 a. Here, it was proposed that the enantioselectivity was enhanced due to employment of the cyclic rigid structure of *(R)‐* L*2 as compared to *(S)‐* L*1 a and the yield was increased due to the higher Lewis acidity of *(R)‐* L*2 than *(S)‐* L*1 a. Using this system, a wide range of chiral amines **4** (89 % *ee*) were accomplished in high yields (up to 99 %) from the enantioselective hydrogenation of imine (Scheme 1b).

**Scheme 2:**
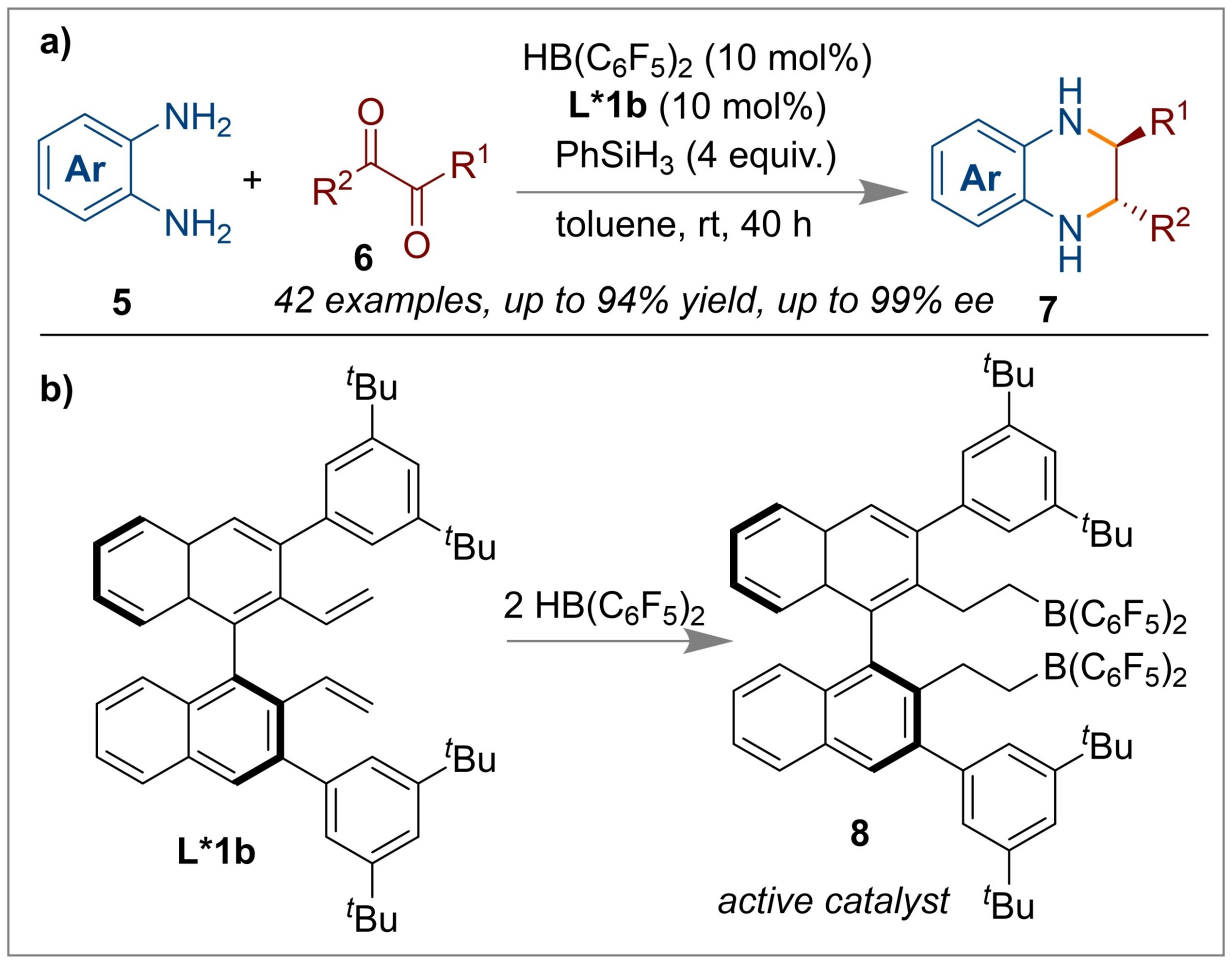
Enantio‐ and diastereoselective synthesis of *trans*‐2,3‐disubstituted‐1,2,3,4‐tetrahydroquinoxalines from the cyclisation of 1,2‐diaminobenzene and 2,3‐butane‐dione.

Recently, the Walsh group has reported the one pot tandem cyclisation of 1,2‐diaminobenzene **5** and 2,3‐butane‐dione **6** to afford highly enantio‐ and diastereoselective *trans*‐2,3‐disubstituted‐1,2,3,4‐tetrahydroquinoxalines **7** (Scheme 2a).^[15]^

**Figure 2:**
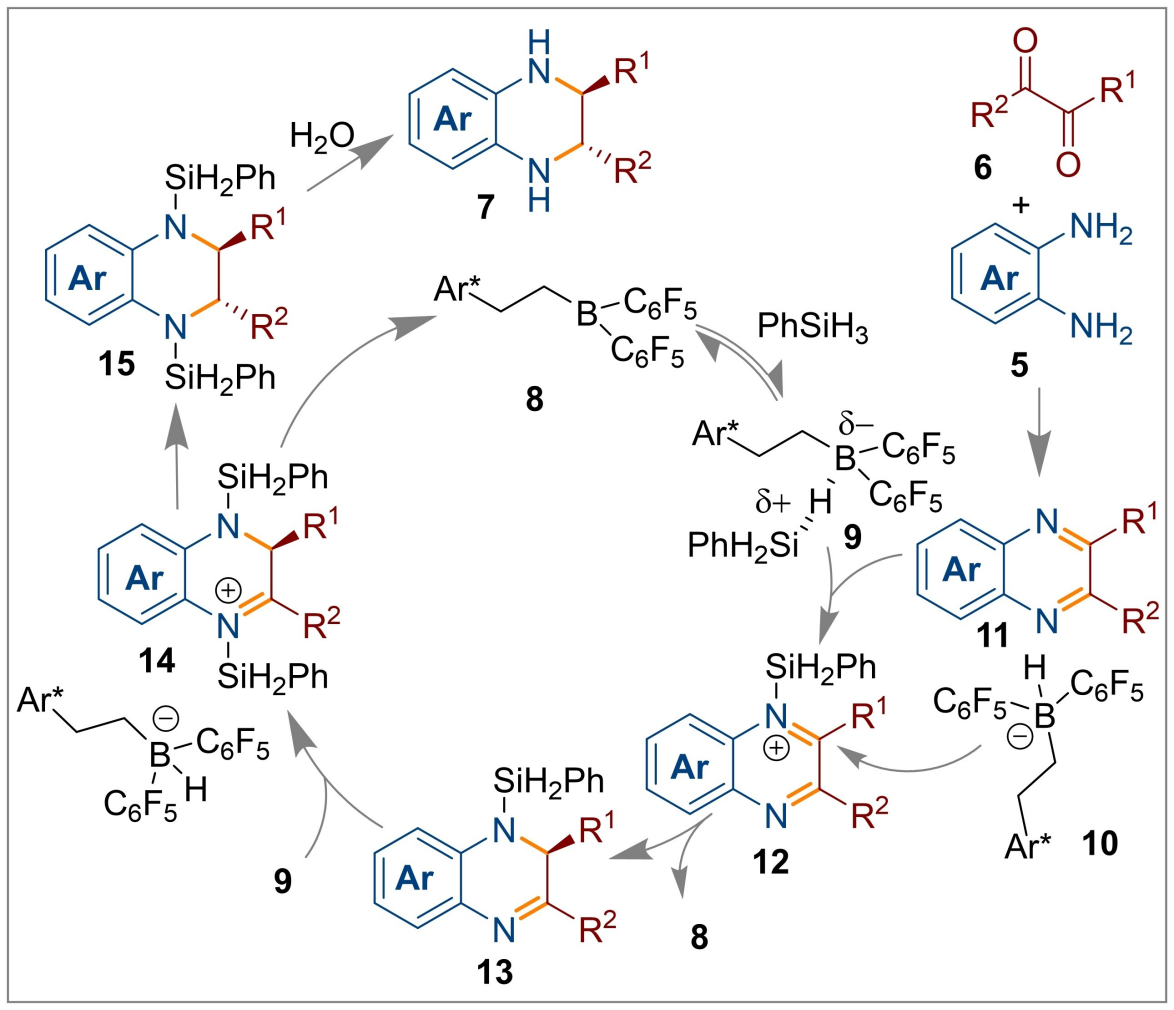
Plausible mechanism for the synthesis of *trans*‐2,3‐disubstituted‐1,2,3,4‐tetrahydroquinoxalines.

The authors claimed that the choice of the hydrosilane reductant and borane catalyst could help in determining the high diastereo‐ and enantioselectivity (>20 : 1 *dr* and 99 % *ee*) observed. The chiral ligand employed (*(R)‐* L*1 b) was a BINOL derived diene similar to that reported by Du which generates the active bis‐borane catalyst **8** in situ following hydroboration with Piers’ borane (Scheme 2b). As shown in the mechanism (Figure 2), the chiral borane catalyst **8** activates phenylsilane to generate the borane‐hydrosilane species **9**. The 2,3‐disubstituted quinoxaline **11** (generated from the 1,2‐diaminobenzene and 1,2‐diketone) then reacts with **9**, generating the ion pair silylated iminium intermediate **12** along with the borohydride anion **10**. Next, intermediate **12** provides the *N*‐silylated dihydroquinoxaline **13** from the borohydride reduction of the activated C=N bond in **12**. This is subsequently converted to the ion pair intermediate **14** with another equivalent of **9** through the same process of **8→9→12** described above. The diastereoselective hydride attack at the C3‐position of the dihydro quinoxalinium moiety furnished the *trans*‐*N*‐silylated‐2,3‐disubstituted‐1,2,3,4‐tetrahydroquinoxaline **15** by regenerating the borane complex **8**. The diastereoselectivity in this step arises due to hydride transfer from the very bulky borohydride and 1,2‐addition to the least hindered face of the C=N bond (on the opposite side to the R^1^ group). Finally, the product **7** was obtained by hydrolysis.

**Scheme 3:**
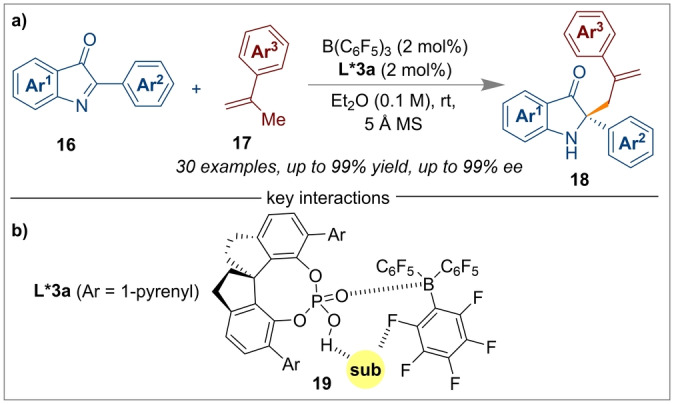
Enantioselective ketimine‐ene reaction using a B(C_6_F_5_)_3_/*(S)‐* L*3 a CPA catalyst.

**Scheme 4:**
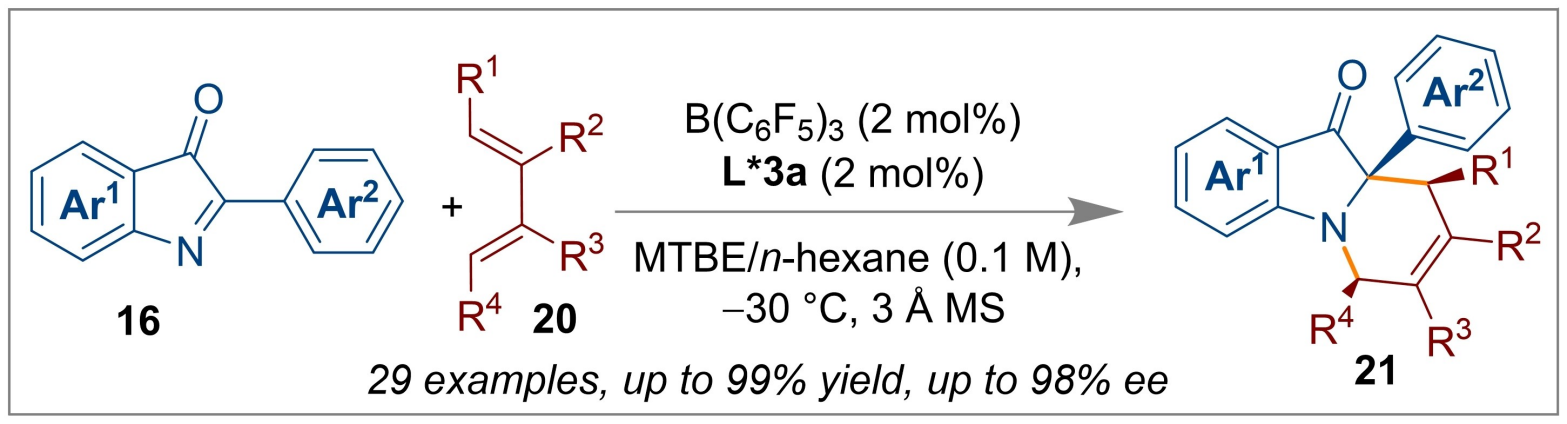
Aza‐Diels–Alder reaction from ketimines using a B(C_6_F_5_)_3_/*(R)‐* L*3 a CPA catalytic system. MTBE=methyl *tert*‐butyl ether.

CPAs are common catalysts, and have found a broad range of applications particularly in enantioselective organocatalysis[^47^, ^48^] and transition metal catalysis.[^49^, ^50^] CPAs have also been used in conjunction with boron Lewis acids for enantioselective catalysis. For example, Li and co‐workers developed a highly enantioselective ketimine‐ene reaction of 2‐aryl‐3*H*‐indol‐3‐ones **16** with α‐methylstyrene **17** using CPA *(R)‐* L*3 a and B(C_6_F_5_)_3_ as a catalytic system, without affecting the carbonyl group present in the substrate (Scheme 3a).^[51]^ The role of the B(C_6_F_5_)_3_ was to increase the overall acidity of the CPA, whilst stabilising the transition state **19** through a H⋅⋅⋅F interaction between the aromatic C−H of the 2‐aryl‐3*H*‐indol‐3‐one substrate and an *o*‐fluorine atom of one of the C_6_F_5_ aromatic rings of B(C_6_F_5_)_3_ (Scheme 3b). Crucially, a H⋅⋅⋅O interaction between the ketimine N−H and the oxygen of the CPA in the transition state was also responsible for enhancing the stereoselectivity of the process. Furthermore, their continuous efforts examining the reactivity of ketimines led to an aza‐Diels–Alder reaction with unactivated dienes **20** using the same B(C_6_F_5_)_3_/*(R)‐* L*3 a CPA catalytic system.^[18]^ A wide array of enantioenriched tetrahydro pyridine derivatives **21** were isolated with excellent yields (up to 99 % and 98 % *ee*) for the [4+2] cycloaddition reaction (Scheme 4).

**Scheme 5:**
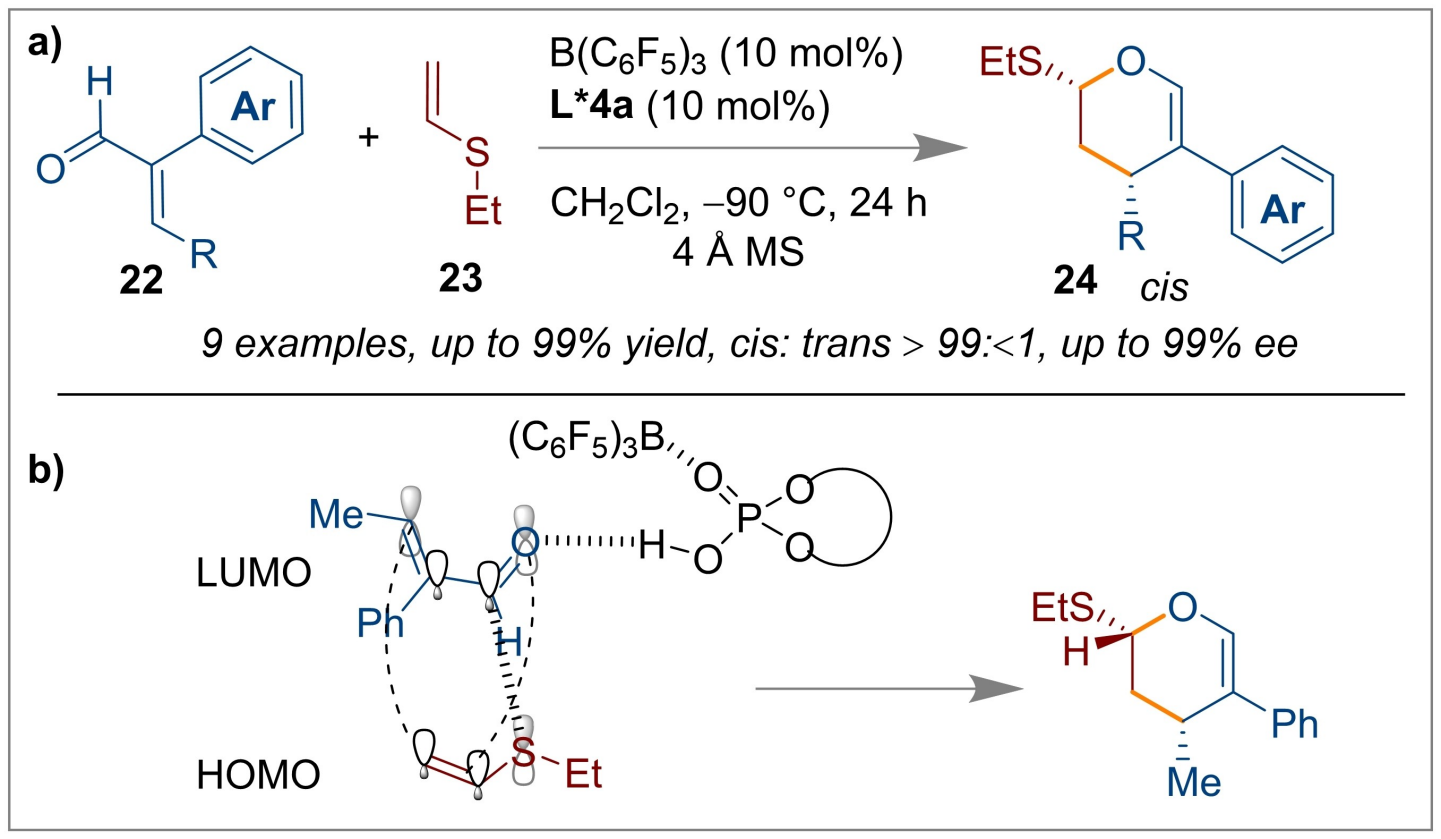
[4+2] Cycloaddition reactions of acroleins and ethyl vinyl sulfides using a B(C_6_F_5_)_3_/*(R)‐* L*4 a CPA catalytic system.

Diels–Alder cycloaddition reactions are an extremely important bond forming tool for the construction of six‐membered carbo/heterocycles.^[52]^ Ishihara and co‐workers have demonstrated an inverse electron demand hetero Diels–Alder reaction of acroleins **22** and ethyl vinyl sulfides **23** similarly using a chiral Brønsted acid/B(C_6_F_5_)_3_ catalytic system (Scheme 5a).^[17]^

**Scheme 6:**
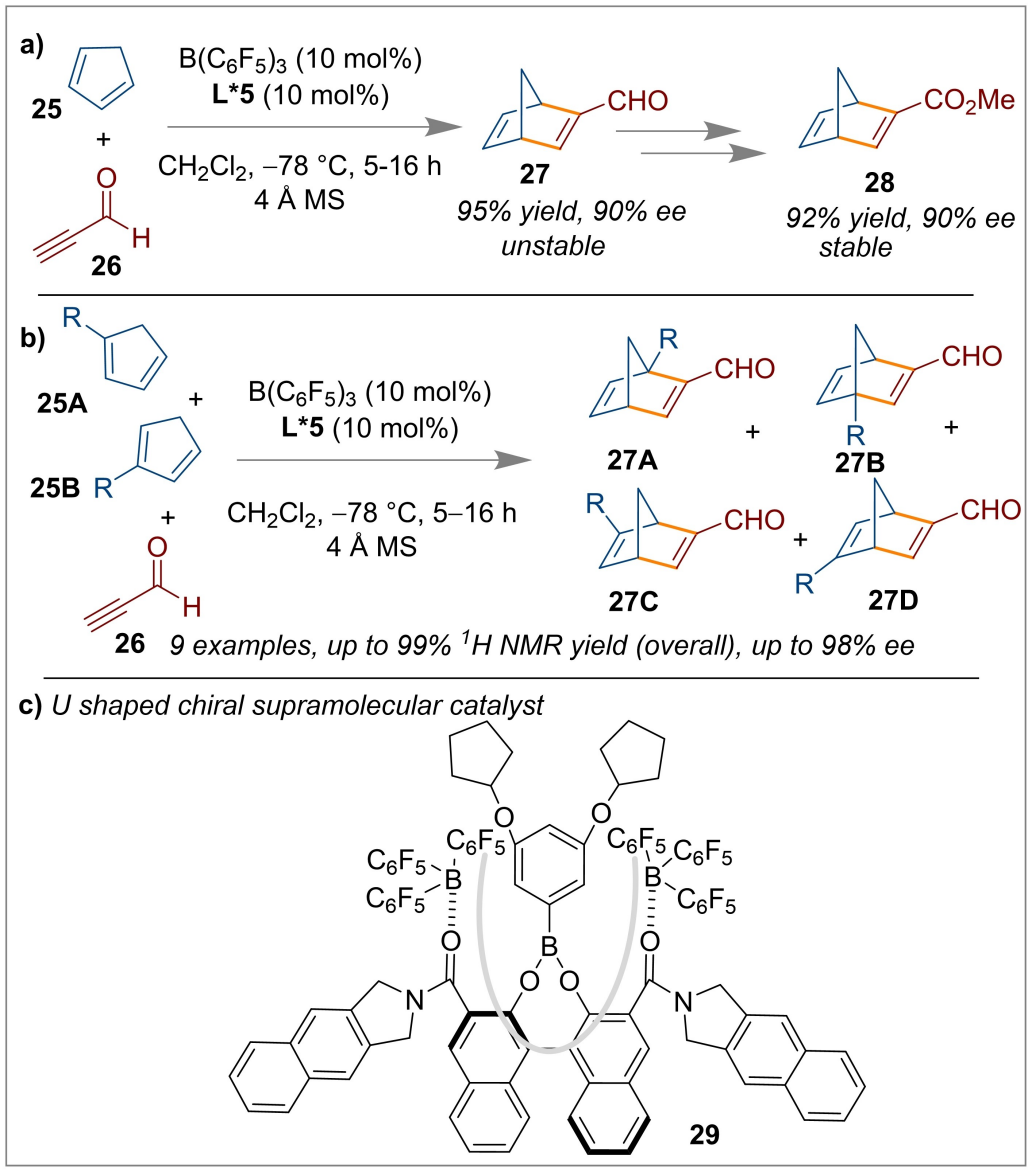
[4+2] cycloaddition reactions using BINOL‐derived *(R)‐* L*5/B(C_6_F_5_)_3_ catalytic system.

The chiral supramolecular Brønsted acid *(R)‐* L*4 a (10 mol %) in the presence of 10 mol % B(C_6_F_5_)_3_ allowed excellent control of both the regio‐ and stereoselectivity for the [4+2] cycloaddition reaction. As indicated in Scheme 5b, the cycloaddition to generate selectively the *cis* product was accelerated by favourable *endo* orbital overlap which afforded enantioenriched 3,4‐dihydro‐2*H*‐pyrans **24**. The secondary orbital interaction was governed by the large lobe of sulfur through a preferred HOMO–LUMO geometry which determined the reactivity and selectivity. Ishihara's group also investigated the selectivity of [4+2] cycloaddition reactions using optically active supramolecular U‐shaped catalysts **29** based on a boron‐containing 3,3‐Lewis‐base‐functionalised chiral BINOL framework with B(C_6_F_5_)_3_ (Scheme 6c).^[53]^ Screening of several derivatives showed *(R)‐* L*5 was the best choice of ligand when combined with B(C_6_F_5_)_3_. This was proposed to give a deep and narrow chiral U‐shaped cavity to allow excellent enantiocontrol in the reaction to generate Diels–Alder products **27** (95 % yield, 90 % *ee*). This product was unstable but could be transformed into more stable and useful chiral compounds for example by oxidation and methylation to form an α,β‐unsaturated ester **28** (Scheme 6a). To understand the substrate‐dependant regioselectivity, a control experiment with a 1 : 1 mixture of 1‐ and 2‐alkyl substituted cyclopentadiene **25 A** and 25 B was employed, and product **27 A** was obtained selectively (Scheme 6b).

**Scheme 7:**
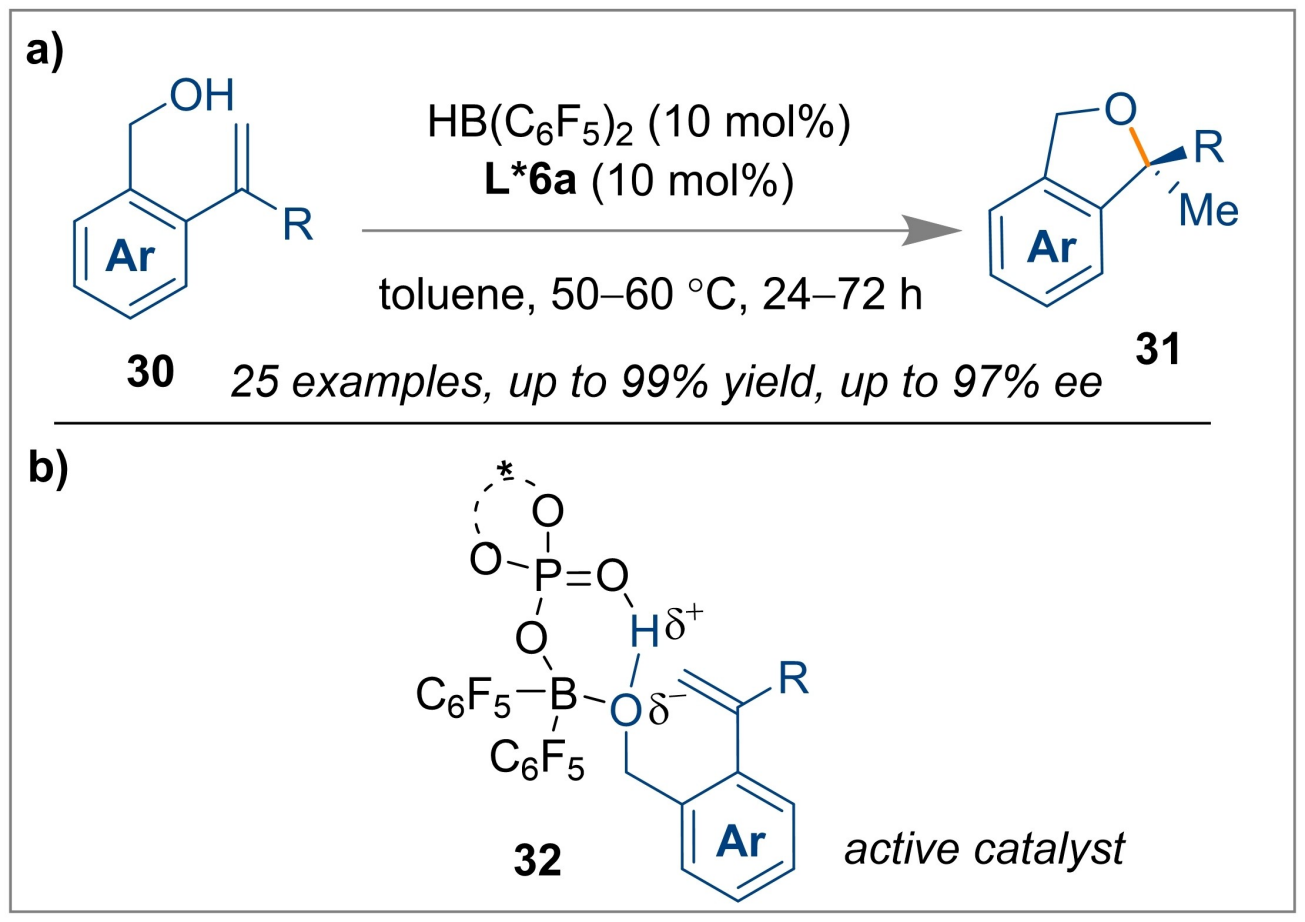
Hydroalkoxylation of alkenes using a chiral boro‐phosphate generated in situ from HB(C_6_F_5_)_2_ and *(R)‐* L*6 a.

Chiral boro‐phosphates are used in asymmetric induction because of their easy accessibility and viable synthetic modification. The Du group implemented a chiral boro‐phosphate catalyst, which was obtained in situ from the reaction of Piers’ borane and CPA *(R)‐* L*6 a through liberation of H_2_ gas, for the intramolecular hydroalkoxylation of alkenes **30** (Scheme 7).^[19]^ As shown in Scheme 7b, the hydroxyl group of **30** is activated by the chiral boro‐phosphate catalyst acting as either a Lewis acid or FLP through the intermediate **32**, which facilitated the intramolecular cyclisation within 2‐vinyl benzyl alcohol **30** to provide optically active 1,3‐dihydroisobenzofurans **31** (up to 94 % *ee*) as cyclic ethers in high yields (up to 99 %).

The implementation of chiral borane catalysis has emerged in the scope of main group catalysed enantioselective reactions as it avoids the use of external chiral ligands which can be expensive or not readily accessible. Additionally, the efficient coordination of achiral boranes with chiral ligands in situ seen above is essential to commence a catalytic cycle. The direct use of chiral borane catalysts may provide more robust catalysts and may remove any initiation period in the reaction.

**Scheme 8:**
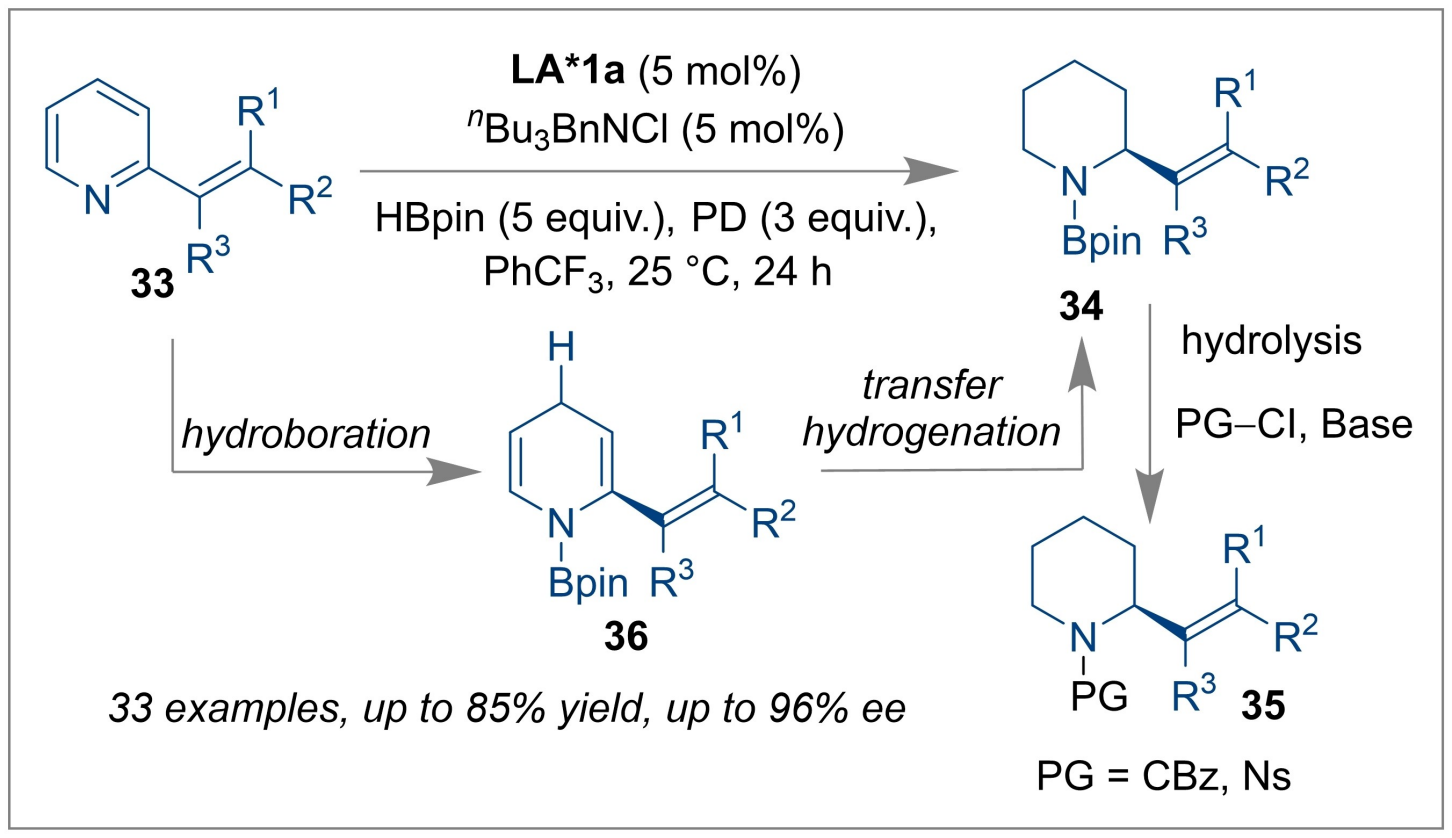
Chemoselective and enantioselective reduction of 2‐vinyl‐substituted pyridines catalysed by a chiral spiro‐bicyclic bis‐borane LA*1 a.

Qiuling et al. have recently reviewed that the asymmetric hydrogenation of various imines, enamines and quinolines could be achieved by utilising several newly designed chiral borane catalysts.^[11]^ Using this methodology, not only the enantioselectivity but also the chemo‐selectivity can be controlled with the chiral borane catalyst. For example, Wang and co‐workers introduced a highly chemo‐ and enantioselective reduction of 2‐vinyl‐substituted pyridines **33** catalysed by a chiral spiro‐bicyclic bis‐borane LA*1 a (Scheme 8).^[54]^ Here HBpin was used as a reducing agent and *N*‐(3,5‐bis(trifluoromethyl)phenyl)acetamide (PD), an acidic amide, was employed as an H^+^ donor. The reaction proceeded through 1,4‐hydroboration followed by transfer hydrogenation via the dihydropyridine intermediate **36**. Subsequently, hydrolysis and base‐mediated N−H protection of piperidine **34** yielded highly enantioenriched N−H protected piperidines **35** with excellent yields. The application of a similar chiral borane was further evaluated by Wang and co‐workers for enantioselective cycloaddition reactions.

**Scheme 9:**
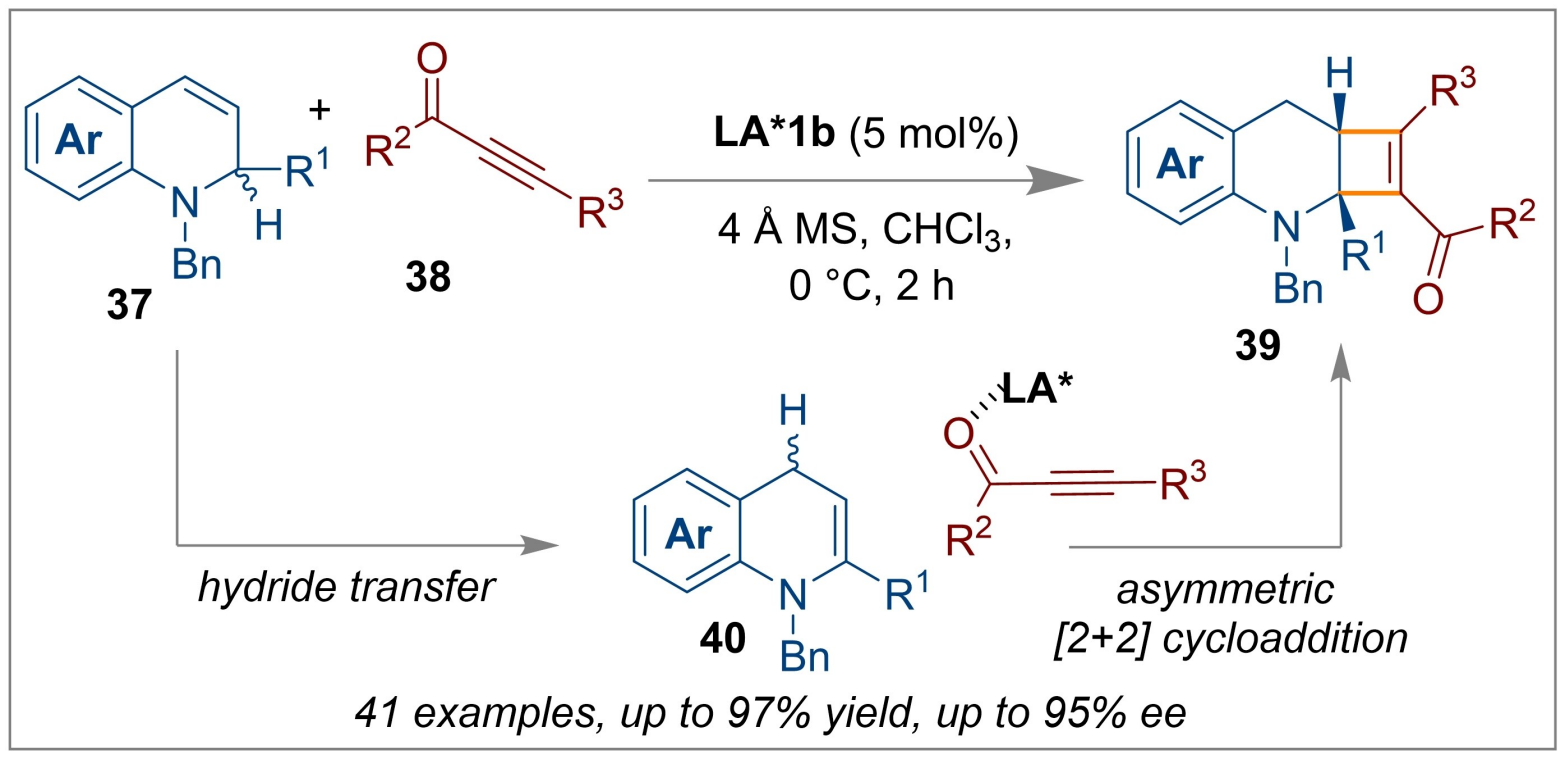
[2+2] cycloaddition reactions of 1,2‐dihydroquinolines and alkynones using chiral spiro‐bicyclic bis‐borane LA*1 b.

Chiral borane LA*1 b was found to act as a bifunctional catalyst. Initially, the catalyst effects the hydride transfer from the 1,2‐dihydroquinoline **37** generating 1,4‐dihydroquinoline intermediate **40**. Subsequently the catalyst promoted the asymmetric [2+2] cycloaddition reaction with an alkynone **38** leading to the facile and atom‐economical one‐pot synthetic route to enantioenriched tetrahydroquinoline‐fused cyclobutenes **39** (Scheme 9).^[55]^

Recently, Chen and Yang made a remarkable contribution to this family as they introduced bis pyrrolidine diboronates (BPDB) as a new class of air and moisture‐stable chiral borane catalysts for the highly regioselective asymmetric exo‐Diels–Alder reactions of mono‐carbonyl based dienophiles.^[56]^

**Scheme 10:**
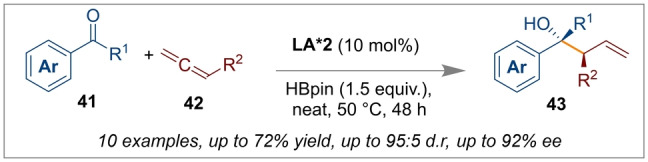
Synthesis of homoallylic alcohol from a chiral boron‐catalysed allylation of ketones.

A further extension on the application of chiral Lewis acidic systems was reported recently by Thomas et al. Chiral Lewis acid LA*2 was found to be effective for *trans*‐borylation reactions to assist carbon‐carbon bond forming reactions. The synthesis of homoallylic alcohols **43** from a chiral boron‐catalysed allylation of ketones **41** with allenes **42** was reported in excellent diastereo‐ and enantioselectivities (95 : 5 *dr* and 92 : 8 *ee* respectively) (Scheme 10).^[57]^

### 2.2 Frustrated Lewis Pair Catalysis using Chiral Lewis Acid or Chiral Lewis Base

**Scheme 11:**
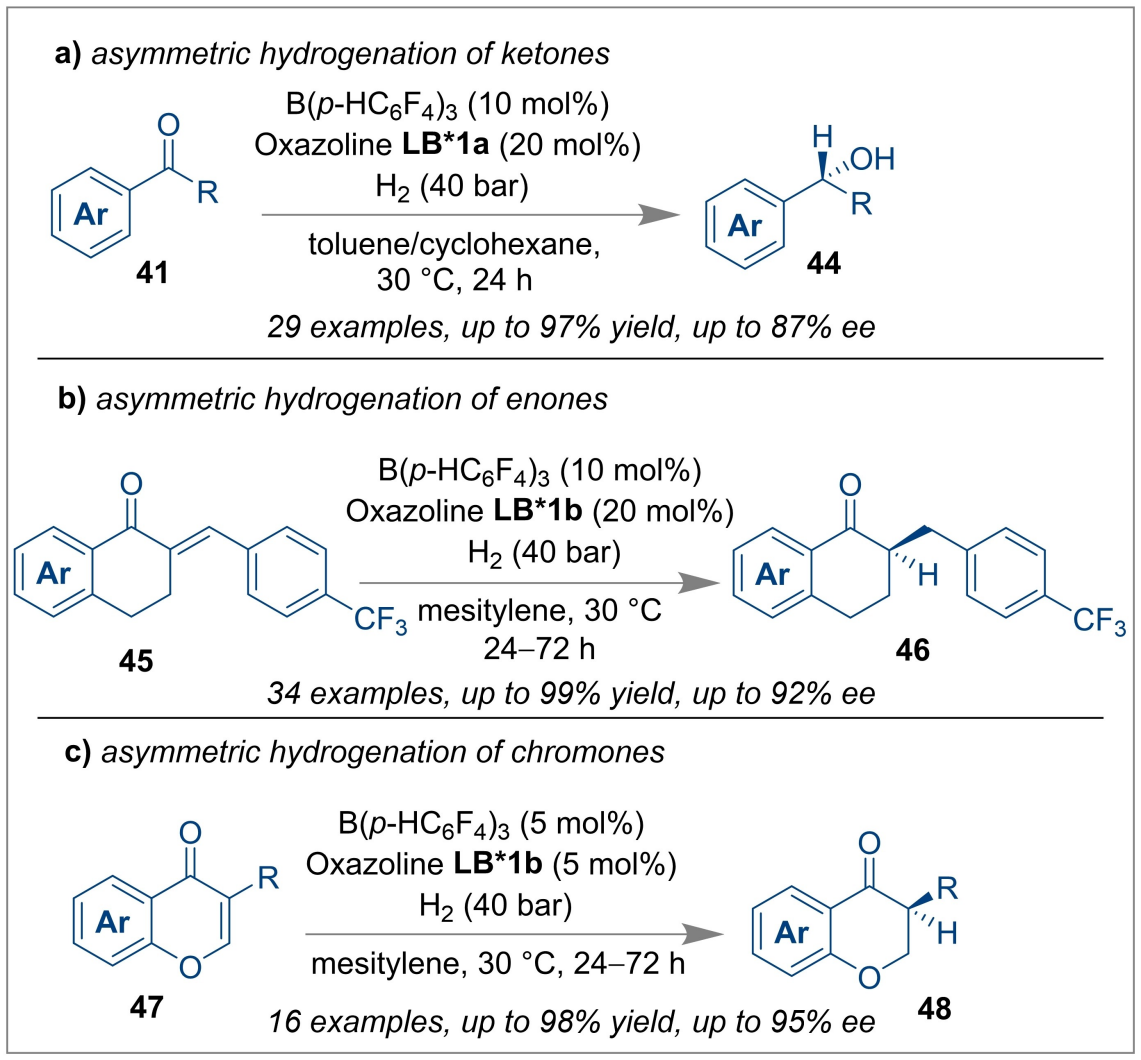
Asymmetric hydrogenation of ketones, enones and chromones using chiral Lewis base derived FLPs.

**Scheme 12:**
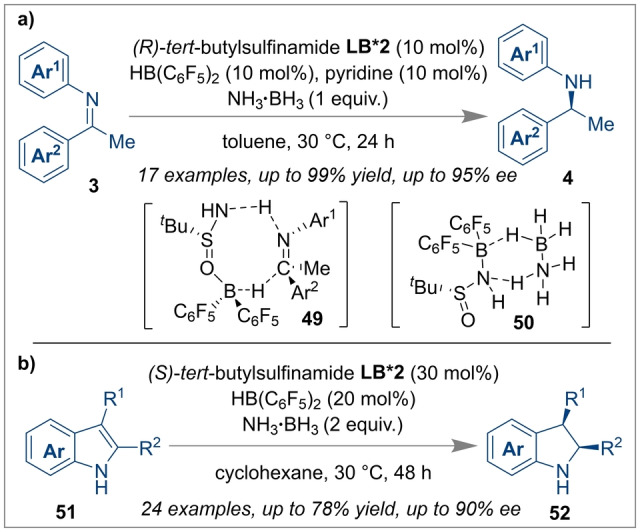
Asymmetric hydrogenation of imines and unprotected indoles.

The unquenched reactivity of FLPs has found widespread applications for heterolytic H_2_ splitting and hydrogenation catalysis.[^58^, ^59^] These key discoveries have prompted the asymmetric reduction of unsaturated substrates using FLPs bearing chiral Lewis acids or bases, or chiral intramolecular FLPs. Several groups including ourselves, Wasa and Du have contributed reviews covering recent developments of enantioselective reactions using FLP catalysts, however, a brief overview of more recent results is reported herein to understand the catalytic activity of stereogenic FLP systems for enantioselective bond forming reactions.[^20^, ^21^, ^22^] The Du group recently disclosed the asymmetric hydrogenation of ketones **41**, enones **45** and chromones **47** using a chiral FLP based on an optically active oxazoline LB*1 as a Lewis base and an achiral borane as a cooperative Lewis acid catalyst.^[60]^ The hydrogenated products (**44**, **46** and **48**) were obtained in very high yields and enantioselectivities (Scheme 11). This work was the first highly successful attempt of using a chiral Lewis base derived FLP for enantioselective hydrogenation of electron‐deficient unsaturated ketones. In another effort to use a chiral Lewis base in FLPs for the enantioselective hydrogenation of imines, Du and co‐workers disclosed the use of achiral Piers’ borane and chiral *tert*‐butylsulfinamide LB*2 as an FLP with ammonia borane as a hydrogen source.^[61]^ The mechanism on how the reaction proceeds entails an 8‐membered cyclic transition state **49** between the FLP components and the substrate **3**, which renders the proton and the hydride addition highly stereospecific, affording chiral amines **4** with yields up to 99 % and enantioselectivities up to 95 % (Scheme 12a). Pyridine was used as an additive to inhibit racemisation by trapping the free Piers’ borane in the reaction system. More recently, the same group extended their work for unprotected indoles **51** (Scheme 12b), where they could isolate chiral indolines **52** with up to 90 % *ee* although the yields were moderate (40–78 %).^[62]^

**Scheme 13:**
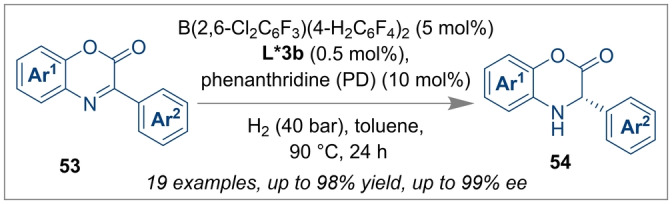
Enantioselective transfer hydrogenation of benzoxazinones using a CPA and borane Lewis acid.

Inspired by Du's work on the enantioselective hydrogenation of 2,7‐disubstituted 1,8‐naphthyridines, Shi et al. reported the enantioselective hydrogenations of *N*‐heterocycles catalysed by a cooperative CPA and a borane/substrate FLP system using ammonia borane as a hydrogen source. However, in this case the observed enantioselectivities were low (up to 29 % *ee*).^[63]^ The idea of using a CPA as a co‐catalyst in FLP catalysed asymmetric hydrogenation reactions was further explored by Du. The highly enantioselective transfer hydrogenation of benzoxazinones **53** was achieved using the CPA *(S)‐* L*3 b with a borane/phenanthridine FLP catalyst. Optically active dihydrobenzoxazinone **54** with up to 99 % *ee* and 98 % yields were obtained (Scheme 13).^[64]^

**Scheme 14:**
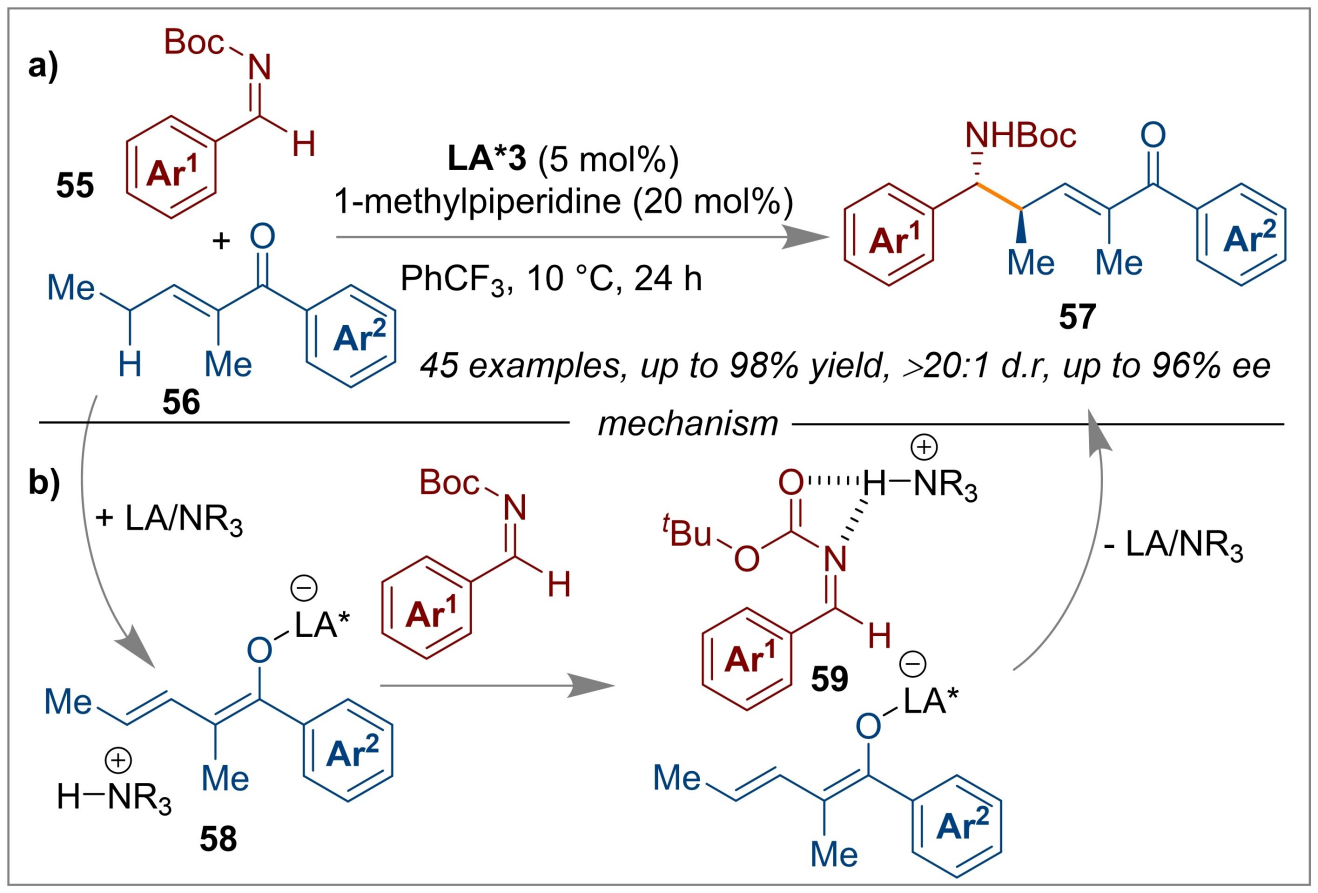
Mannich addition of acyclic α,β‐unsaturated ketones to imines using a chiral Lewis acid based FLP.

Apart from hydrogenation catalysis, FLPs have found other catalytic applications in enantioselective synthesis. Wang and co‐workers employed the use of a Lewis acidic chiral bicyclic bisborane LA*3 and 1‐methylpiperidine as an achiral Lewis base to promote a highly enantioselective vinylogous Mannich addition of acyclic α,β‐unsaturated ketones **56** to imines **55** (Scheme 14a).^[65]^ The reaction proceeds through the cooperative action of the LA*3, which upon coordination of the ketone moiety increases the acidity of the γ‐proton which is then abstracted by the Lewis base, giving rise to a formal ammonium enolate **58**. The Brønsted acid derived from the ammonium salt activates the imine in **59**, favouring the Mannich addition (Scheme 14b). Using this protocol, several δ‐amino‐α,β‐unsaturated carbonyl derivatives **57** can be synthesised in high yields and high enantioselectivities.

**Scheme 15:**
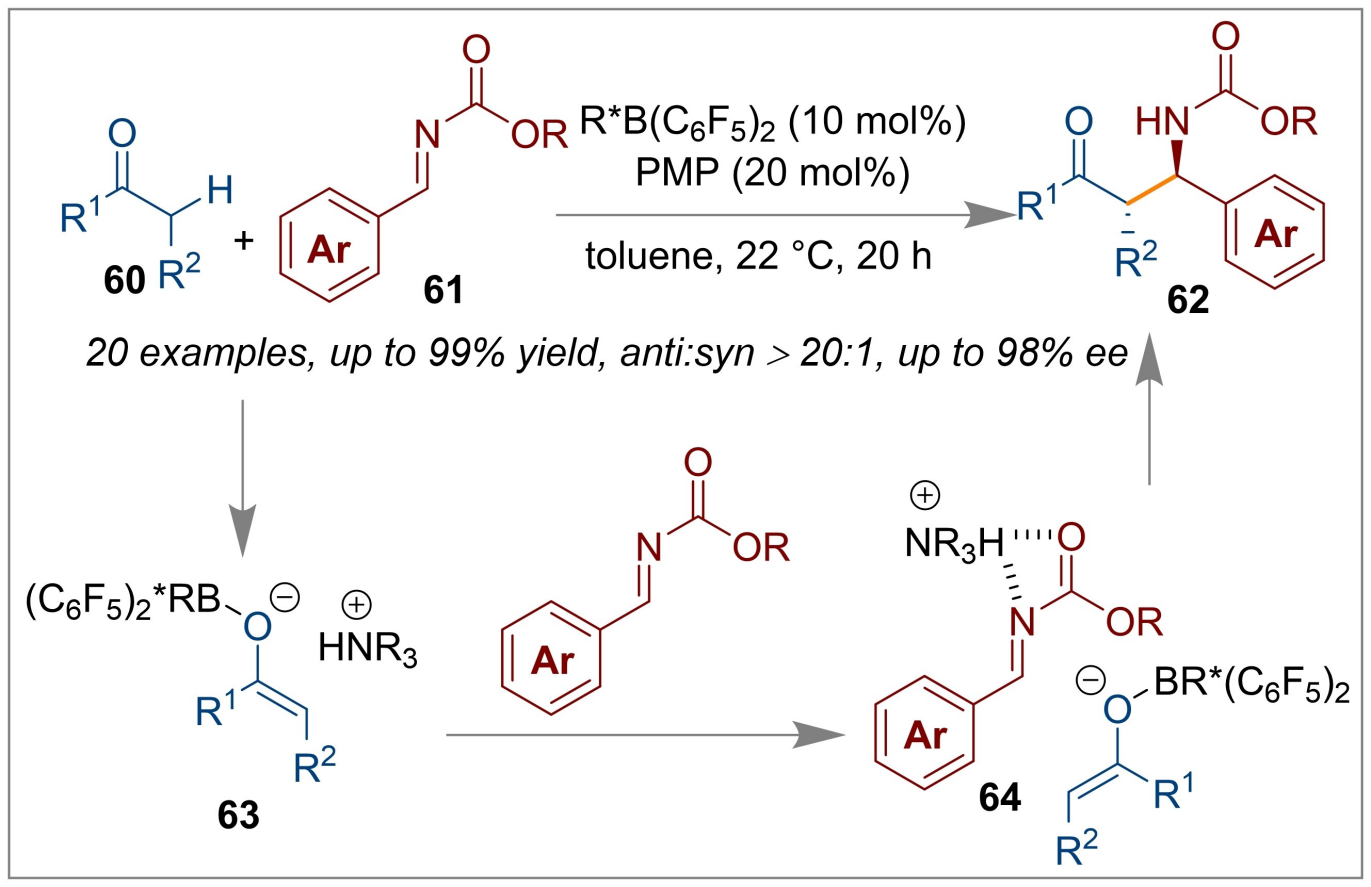
Asymmetric Mannich addition between ketones and boc‐protected imines using a chiral Lewis acid derived from BINOL and an achiral Lewis base.

Similarly, Wasa catalysed the asymmetric direct Mannich addition between ketones **60** and imines **61** employing a chiral BINOL derived Lewis acid and an achiral Lewis base (Scheme 15).^[66]^ In these reactions the most active chiral boranes were synthesised from the double hydroboration of BINOL derived (*R)‐* L*1 c and (*R)‐* L*1 d to give the active chiral Lewis acid component of the FLP [R*B(C_6_F_5_)_2_]. Pentamethylpiperidine (PMP) was utilised as the Lewis basic component of the FLP. During the investigation of a suitable chiral organoborane, the authors observed that the stereo‐electronic effect of the 3,3′‐substituents on the BINOL scaffold were crucial to ensure both high reactivity and enantioselectivity. With this methodology, Mannich addition products **62** from ketones, esters and thioesters with boc‐protected imines were achieved in quantitative yields (up to 99 %) and with excellent enantioselectivities, via the intermediates **63** and **64**. Crucially, boc‐ protection seems to have a central role in the asymmetric induction presumably due to hydrogen bonding, since the authors observed a deteriorating effect on the enantioselectivity when Ts‐protected amines were used instead.

**Scheme 16:**
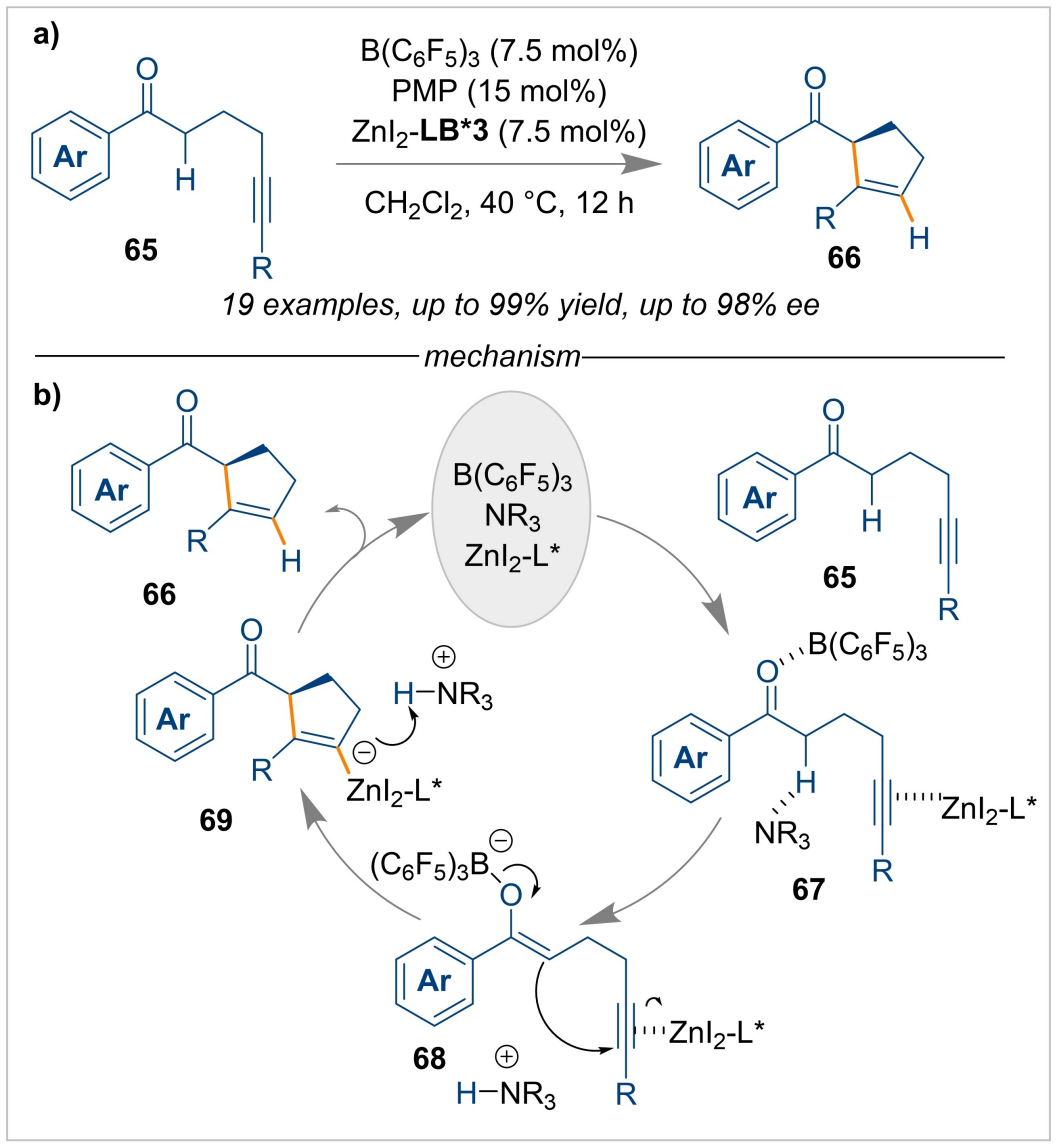
Conia‐Ene type intramolecular cyclisation of γ‐alkynyl ketones using a cooperative FLP/L*ZnI_2_ catalytic system.

The generation of enolates by the cooperative action of FLPs has been mainly employed with acid‐sensitive electrophiles like DEAD (diethyl azodicarboxylate) or boc‐protected imines, which can be activated by the poor Brønsted‐acidic ammonium salts derived from the Lewis basic component of the FLP.[^66^, ^67^, ^68^] In 2019, Wasa disclosed a Conia‐Ene type intramolecular cyclisation reaction of γ‐alkynyl ketones **65** using an FLP system composed of B(C_6_F_5_)_3_ as the Lewis acid and PMP as the Lewis base, with a Zn‐based co‐catalyst bound by chiral bisoxazoline (BOX)‐type ligand (*S,S)‐* LB*3 (Scheme 16a).^[69]^ The mechanism underpinning this reaction consists of the enolate formation by the FLP, and activation of the alkyne by the Zn co‐catalyst (intermediates **67** and **68**), ensuring a 5‐*endo‐dig* cyclisation in an enantioselective fashion. The ammonium salt then protonates the metallate intermediate **69** to complete the catalytic cycle (Scheme 16b).

### 2.3 Aluminium Catalysis using Chiral Al‐Salen Complexes

**Scheme 17:**
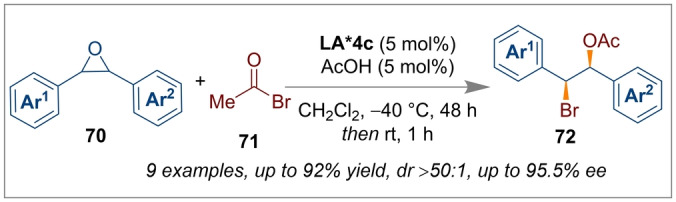
Enantioselective acetyl bromohydrins from achiral epoxides and acetyl bromide using a chiral Al‐salen complex LA*4 c.

Moving down group 13, the element aluminium has also been used in enantioselective catalysis. Unlike boron, aluminium can adopt higher coordination numbers and has been found to readily form complexes with multidentate chiral ligands.^[30]^ In this regard, one common ligand framework is the tetradentate C_2_‐symmetric salen ligand LA*4.^[30]^ During our literature survey on aluminium catalysed enantioselective reactions, it was found that Peters and co‐workers have reported Al‐salen complexes as chiral catalysts for a range of asymmetric transformations. This includes the asymmetric hydroboration of ketones,^[25]^ desymmetrisation of meso‐epoxides,^[28]^ and cyanation of aldehydes[^26^, ^27^] or aldimines.^[29]^ In addition, they have utilised chiral Al‐salen complex‐onium/ammonium salts LA*4 a–**d**. In 2017, Peters reported that achiral epoxides **70** with acetyl bromide **71** could be converted to enantiopure acetyl bromohydrins **72** with reasonable yields using Al‐salen complex *(R,R)‐* LA*4 c (Scheme 17).^[28]^

**Scheme 18:**
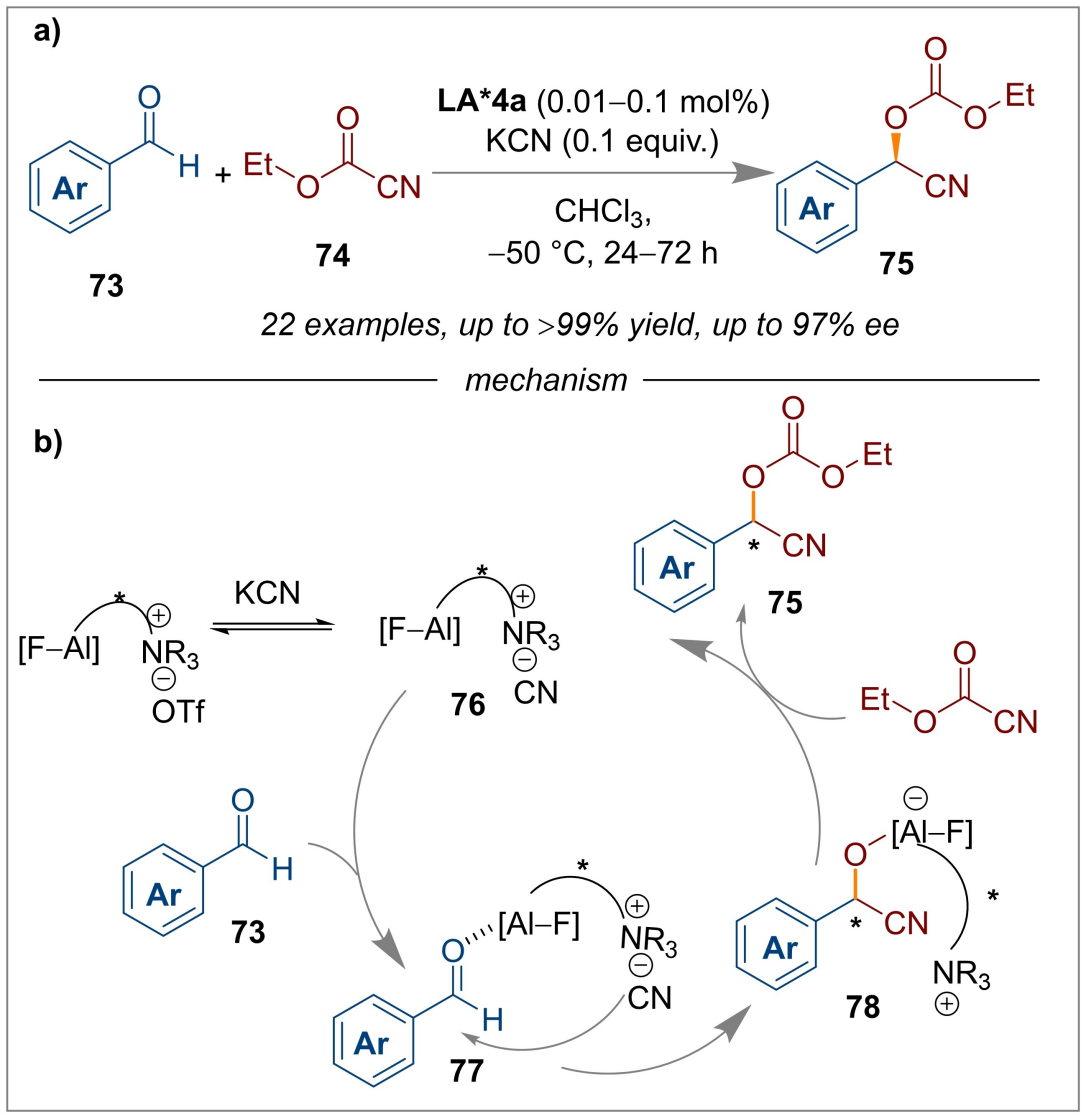
Carboxycyanations of aldehydes using an Al‐F salen complex *(S,S)‐* LA*4 a.

**Scheme 19:**
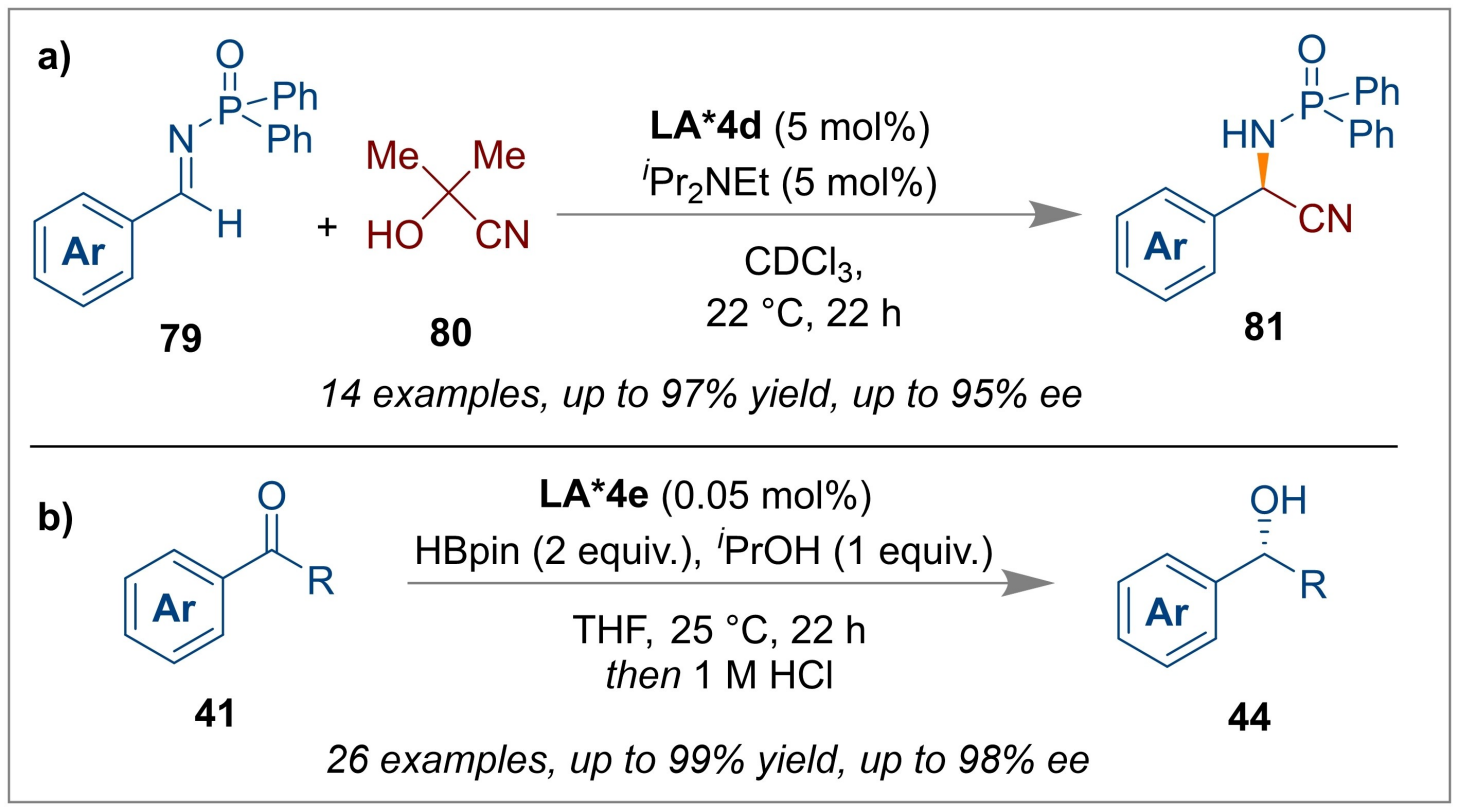
Strecker and hydroboration reactions using chiral Al‐salen complexes *(S,S)‐* LA*4 d and *(S,S)‐* LA*4 e.

Following this, asymmetric carboxycyanations were also reported by the same research group.[^26^, ^27^] The chiral aluminium‐fluorine based salen complex *(S,S)‐* LA*4 a was found to be an excellent catalyst for the carboxycyanations of aldehydes **73** with high enantioselectivities (97 % *ee*) and excellent yields (Scheme 18a).^[27]^ The mechanism depicted in Scheme 18b, shows how the additive KCN (potassium cyanide) helps in improving the reactivity of the Al−F salen complex *(S,S)‐* LA*4 a by an ion exchange process. Nucleophilic addition of the cyanide ion to the carbonyl, in a step referred to as “quasi‐intramolecular” by the authors (indicating temporary or partial separation of the nucleophile ion during the transfer), was found to be driven by coordination of the Al−F complex with the oxygen of the aldehyde **77**. Finally, carboxylation and regeneration of the catalyst was facilitated by ethyl carbonocyanidate. A Strecker type reaction using a cooperative Brønsted base and a salen complex as a Lewis acid *(S,S)‐* LA*4 d has also been developed (Scheme 19a).^[29]^ The reaction performed with *N*‐phosphinoyl aldimines **79** and acetone cyanohydrin **80** gave excellent yields and enantioselectivities of the products **81** that could be used to synthesise enantioenriched α‐amino acids.

The enantioselective hydroboration of ketones is known with borane catalysts,^[70]^ however, the use of Al‐salen complexes instead such as *(S,S)‐* LA*4 e exhibit excellent yields and enantioselectivities in addition to high turnover numbers (TON=15400). The high TON, found to be 1.5–3 orders of magnitude higher than other asymmetric hydroboration catalysts, results from the readily recyclable nature of the catalyst, which is stable during the catalytic cycle (Scheme 19b).^[25]^ In this carbonyl reduction process, HBpin was employed for a hydroboration step and ^*i*^PrOH was used for the proton source in the final alcohol product **44**.

**Scheme 20:**
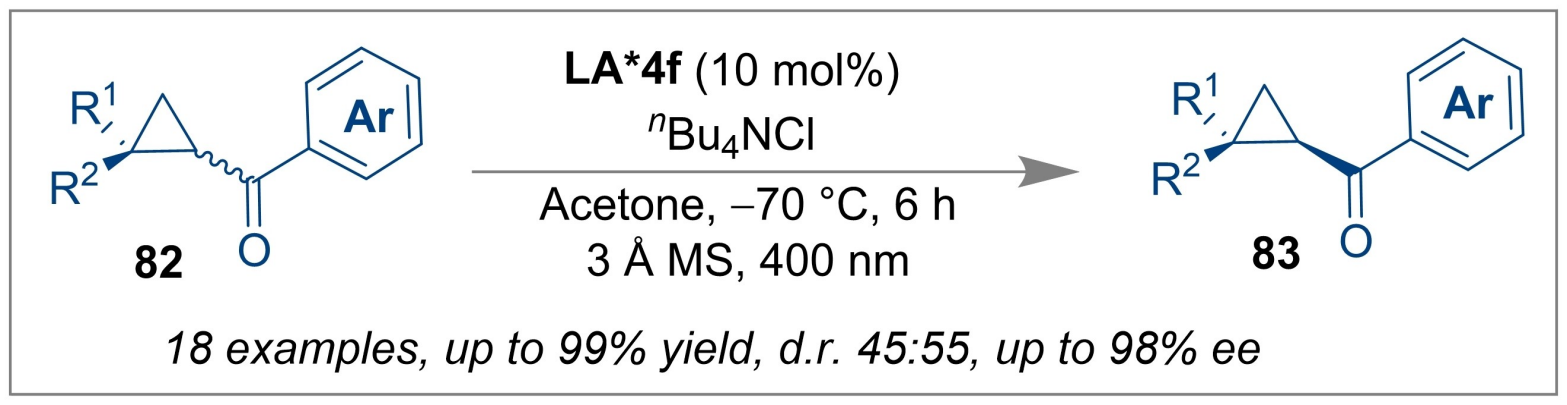
Chiral Al‐salen complex as a visible light photocatalyst for deracemisation of cyclopropyl ketones.

Very recently, Gilmour and co‐workers used the Al‐salen complex *(R,R)‐* LA*4 f as an efficient photocatalyst for the deracemisation of cyclopropyl ketones **82** (Scheme 20).^[71]^ This unique reactivity mode of the Al‐salen complex under λ=400 nm irradiation could transform racemic cyclopropyl ketones into enantioenriched cyclopropyl ketones **83** with a 96 % *ee*. A feasible electron transfer (EnT) process from the excited‐state catalyst (*E*_1/2_(Al*/Al^+^)≈−1.47 V versus SCE) to the racemic cyclopropyl ketone (R^1^=R^2^=Ph, Ar=Ph) (*E*_1/2_=−1.96 V versus SCE) was responsible for the deracemisation reaction. It is anticipated that the implementation of the chiral Al‐salen complex as a visible light photocatalyst will expand the library of main group catalysed enantioselective transformations and would encourage the design of new chiral transition metal‐free photocatalysts.

### 2.4 Bismuth Catalysis using Chiral Phosphoric Acids

**Scheme 21:**
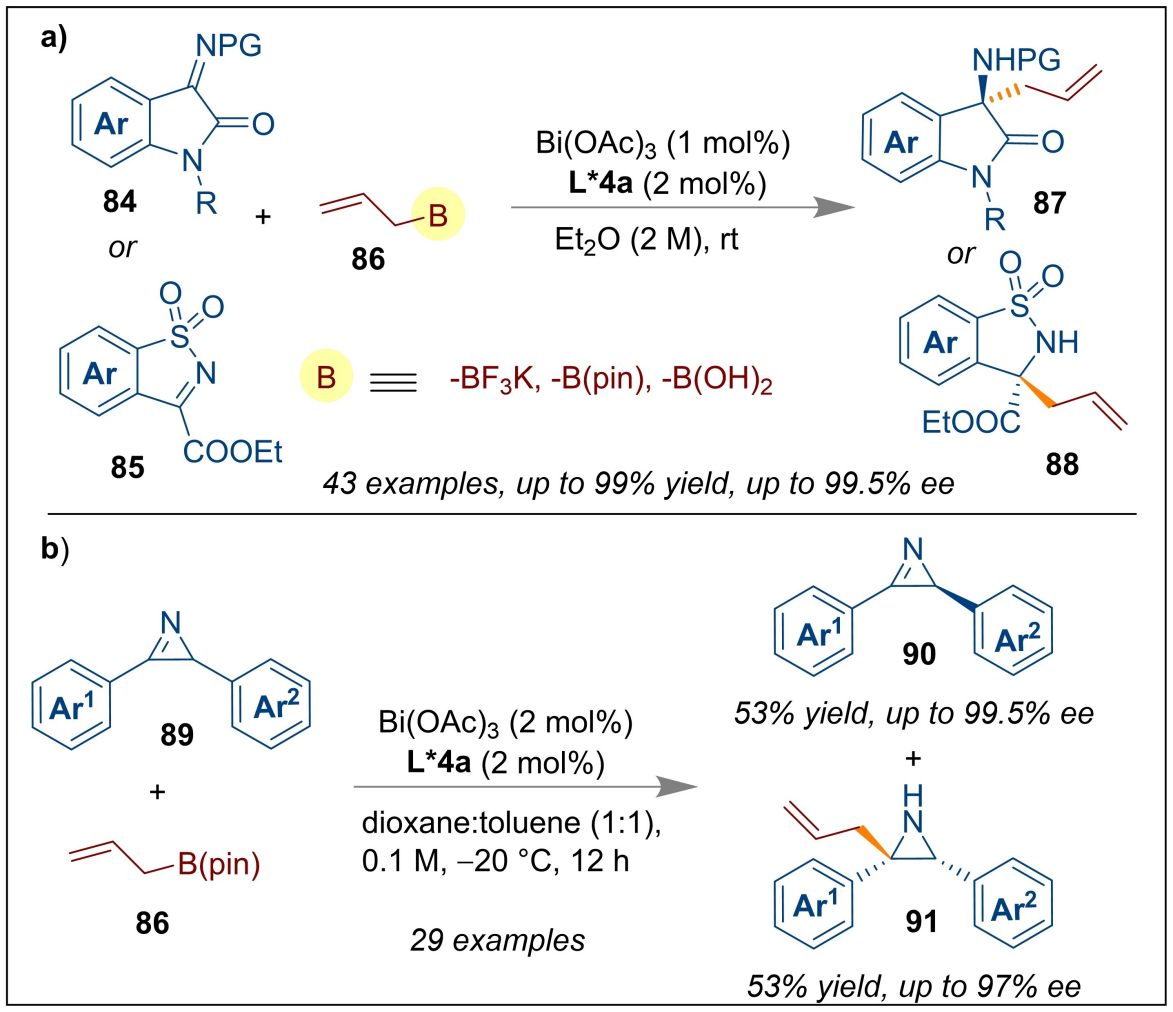
Enantioselective allylation of ketimines and azirins using CPA *(S)‐* L*4 a and Bi(OAc)_3_.

Bismuth catalysis has garnered interest in recent years,^[31]^ and naturally an extension of this field to include enantioselective catalysis has recently been reported. With similarity to the boron Lewis acid catalysis seen earlier, bismuth in combination with CPAs have been reported for stereoselective reactions. In this regard, Li and co‐workers introduced the enantioselective allylation of ketimines **84** and **85** using allyl boronates **86** as an allyl source using Bi(OAc)_3_ and CPA *(S)‐* L*4 a as a catalytic system (Scheme 21a).^[36]^ Twice the amount of CPA was used relative to Bi(OAc)_3_ because the CPA was involved as both an anionic and neutral ligand during the ligand exchange process with Bi(OAc)_3_. The excellent yields (up to 99 %) and high enantioselectivities (up to 99.5 % *ee*) were achieved for a wide variety of chiral 3‐allyl 3‐amino oxindoles **87** and **88**. The developed protocol was applied to the synthesis of (+)‐AG‐041R as a gastrin receptor antagonist, and the total synthesis of (−)‐psychotriasine as naturally occurring bioactive molecules. The same group also reported the highly enantioselective allylation of azirins from a racemic mixture of (2*H*)‐azirines **89** using the same catalytic system (Scheme 21b).^[72]^ As described by the authors, the Bi‐CPA catalyst first bound to the allyl species **86** which then reacted with the racemic azirins at different rates resulting in excellent kinetic resolution (*s*‐factor of up to 127).

**Scheme 22:**
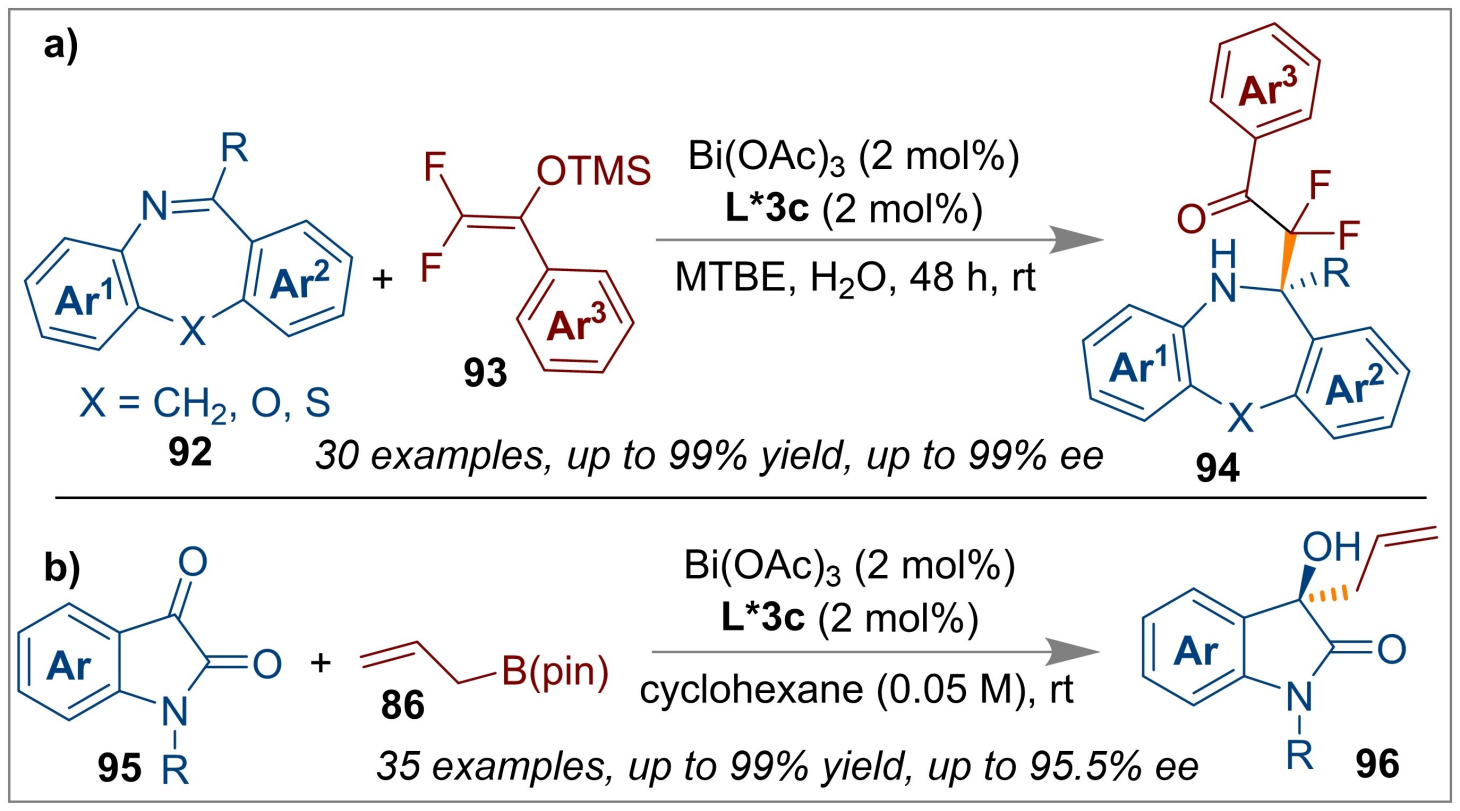
Difluorocarbonylation of dibenzo[b,f][1,4]oxazepanes (top) and allylation reaction of isatins (bottom) using a Bi(OAc)_3_/*(S)‐* L*3 c CPA catalyst.

They also extended their studies to non‐activated imines using a Bi(OAc)_3_/*(S)‐* L*3 c CPA system for the synthesis of a series of dibenzo[b,f][1,4]oxazepines with high yields (up to 99 %) from the enantioselective difluorocarbonylation of dibenzo[b,f][1,4]oxazepane **92** (Scheme 22a).^[37]^ Difluoroenoxysilane **93** was used as a difluorocarbonyl source, however, other mono‐fluorinated silyl enol ethers were also effective. The stereoselectivity of the major isomer was controlled by the non‐covalent lone pair‐π interaction between a fluorine atom in the silyl enol ether and an aromatic ring of the dibenzo[b,f][1,4]oxazepane. The other fluorine atom of the silyl enol ether was involved in F‐hydrogen bonding interactions with an aromatic C−H of the dibenzo[*b,f*][1,4]oxazepane and was also an additional key factor for the formation of the enantioenriched product (99 % *ee*). Li and co‐workers have also investigated the enantio‐ and regioselective allylation reaction of isatins **95** with allylboronates using the same Bi(OAc)_3_/*(S)‐* L*3 c CPA catalytic system to obtain enantioenriched 3‐allyl‐3‐hydroxyoxindoles **96** with excellent yields (Scheme 22b).^[35]^ The authors attributed the high selectivity due to π⋅⋅⋅π interactions between the CPA ligand and the protecting group on the isatin.

**Scheme 23:**
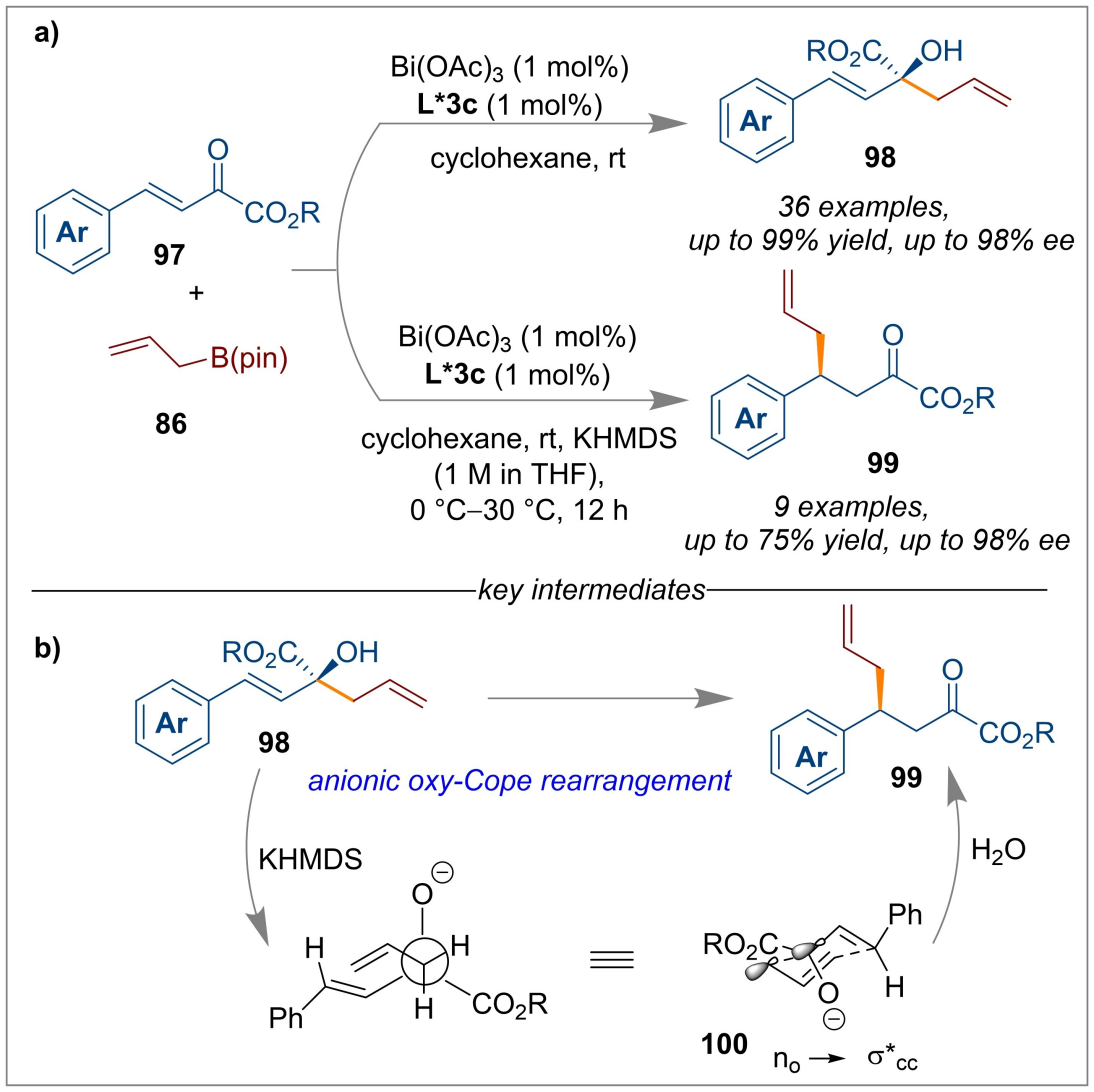
Enantioselective 1,2‐allylation of unsaturated α‐ketoesters using a Bi(OAc)_3_/*(S)‐* L*3 c CPA catalyst.

The combination of Bi(OAc)_3_/*(S)‐* L*3 c CPA for enantioselective transformations has also been extended to α‐ketoesters **97** (Scheme 23a).^[34]^ Reaction occurred at the keto group in the absence of KHMDS (potassium bis(trimethylsilyl)amide) but, when KHMDS was added, the formation of a highly enantioenriched 1,4‐allylation product **99** was obtained through an anionic oxy‐Cope rearrangement. A one‐pot synthetic route accessed either tertiary homoallylic alcohols **98** or γ‐allyl‐α‐ketoesters **99** with both high enantioselectivity (98 % *ee*), and in good to excellent yields (up to 99 % and 75 % yields, respectively). Notably, various α‐ketoesters including important bioactive functionalities such as cholesterol were compatible with this mild Bi‐catalysis approach. The authors proposed that the anti‐HIV agent Tipranavir and nucleoside analogues can be derived from the 1,2‐allylation products. A DFT study into the mechanism also confirmed the origin of an oxy‐Cope rearrangement. In the most stable chair‐like transition state **100** as shown in Scheme 23b, an effective overlap between the nonbonding orbital of the oxyanion (n_o_) and antibonding molecular orbital of the C−C (σ*_C−C_) bond led to the weakening of the C−C bond and accelerated the [3,3]‐rearrangement.

**Scheme 24:**
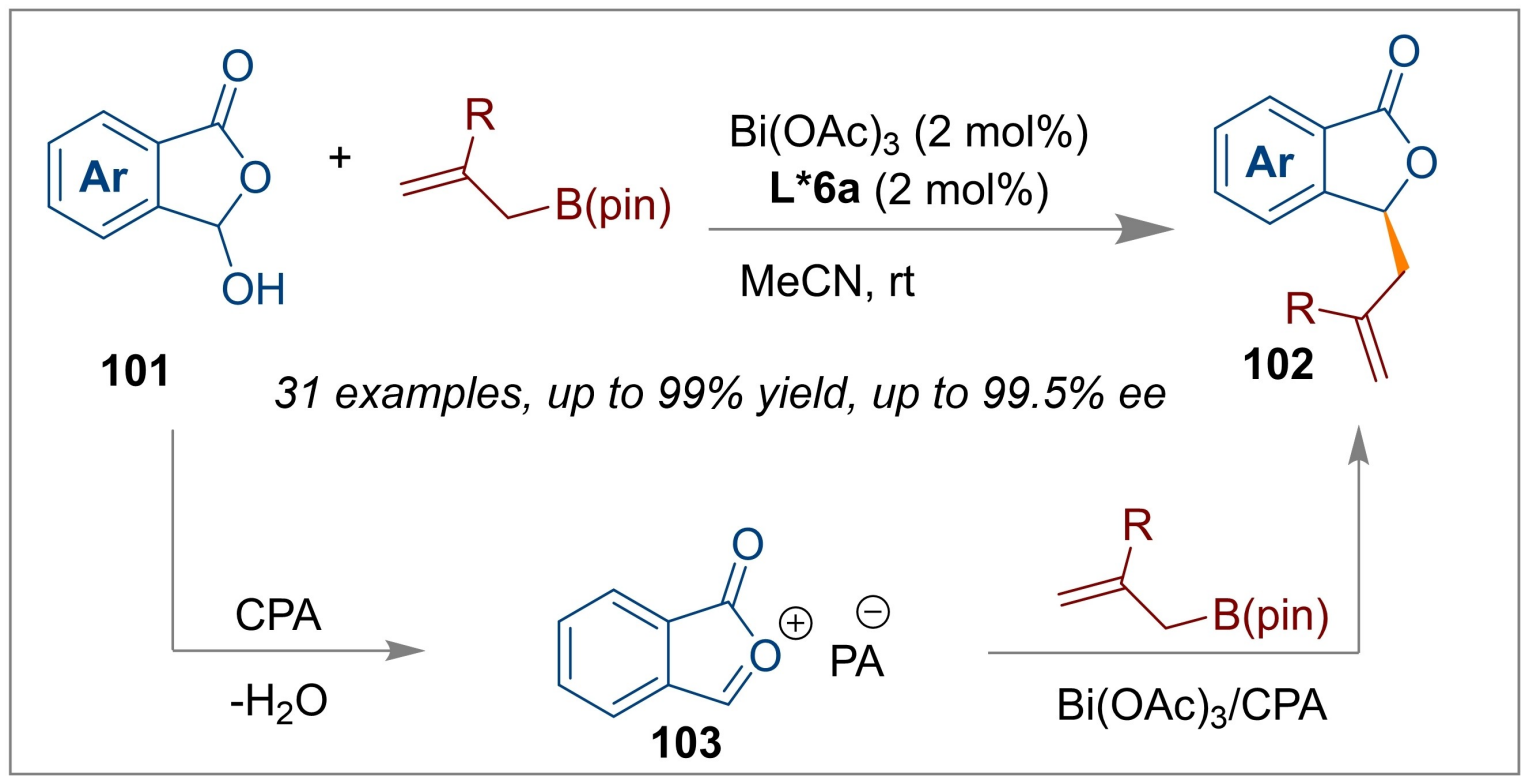
Allylation of 3‐hydroxyisobenzofuran‐1(3*H*)‐ones using Bi(OAc)_3_/*(R)‐* L*6 a catalysis.

Chiral phthalides are important structural motifs found in many natural drugs and biologically active molecules. After the numerous successes using Bi(OAc)_3_/*(S)‐* L*3 c CPA as synergistic catalysts, Li et al. once again introduced a method for the stereogenic allylation of 3‐hydroxyisobenzofuran‐1(3*H*)‐ones **101** (Scheme 24).^[32]^ This time BINOL derived CPA *(R)‐* L*6 a worked best and facilitated the dehydration of the starting material to generate an oxocarbenium ion intermediate **103**. Then the synergistic effect of the Bi(OAc)_3_/CPA catalytic system activated the allylboronate ester which reacted stereo‐specifically with the oxocarbenium ion to produce chiral phthalide derivatives **102**. This exciting protocol could offer a potential synthetic route for synthesising enantioenriched bioactive molecules such as 3‐butylphthalide, (−)‐Herbaric acid and (−)‐spirolaxine.

## 3 Conclusions and Outlook

In conclusion, we have summarised the recent use of p‐block elements in asymmetric catalysis for hydrogenations, allylation of imines and ketones, ketimine‐ene reactions, cycloadditions, Strecker‐type reactions, difluorocarbonylation and desymmetrisation of meso‐epoxides. Both chiral BINOL‐derived unsaturated ligands and CPAs have dominated this field by binding synergistically with boron or bismuth Lewis acids to act as chiral catalysts. The resulting chiral catalysts, typically generated in situ, have been employed for elegant transformations with high yields and excellent enantioselectivities. In addition, chiral FLP systems originated from various Lewis acidic fluorinated aryl boranes and chiral Lewis/Brønsted bases, chiral boranes, and chiral Al‐salen complex have aided the advancement of p‐block enantioselective catalysis. Despite many advances in this field, main group enantioselective catalysis is still relatively new and lags behind that of the more established areas of both transition metal and organocatalysis. We foresee that significant developments in this field will be the focus of research efforts in the future in order to design superior main group catalysts, as well as to advance the scope of enantioselective transformations that can be performed with these elements.

## Conflict of interest

The authors declare no conflict of interest.

4

## Biographical Information

*Dr. Milan Pramanik obtained his MSc from IIT Madras, India in 2016. In 2022, he completed his PhD at NISER Bhubaneswar (India) under the guidance of Prof. Mal. In December, 2022, he joined Prof. Melen's group at Cardiff University (UK) as a postdoctoral researcher. He is the recipient of an Outstanding Doctoral Student Award (2022), and a CRS Young Scientist Outstanding Research Award (2022). His research work in the Melen group focuses on synthetic methodology using borane catalysts*.

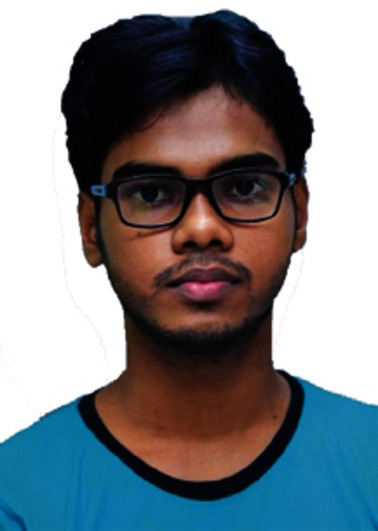

## Biographical Information

*Michael Guerzoni obtained his MSc (cum laude) in chemistry (curriculum “Synthetic Methodologies and Bio‐Organic Chemistry”) in 2020 from the University of Bologna (Italy) working under the supervision of Prof. A. Tolomelli on the organocatalytic synthesis of chiral isoxazolidines. In January 2021, he accepted a PhD position in the groups of Prof. Melen and Dr Richards at Cardiff university (UK). His research focuses on the development of new synthetic methodologies employing boron Lewis acids*.

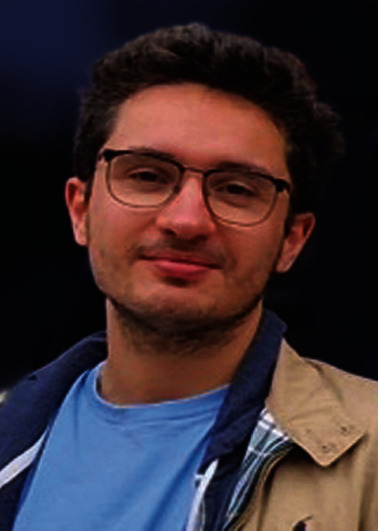

## Biographical Information

*Dr. Emma Richards is a senior lecturer at Cardiff University and co‐director of the Electron Paramagnetic Spectroscopy research group. Her research involves utilising Electron Paramagnetic Resonance to investigate electron‐transfer processes in hetero‐/homogeneous catalysis. Emma is coauthor of the popular Oxford University Press textbook “Electron Paramagnetic Resonance”*.

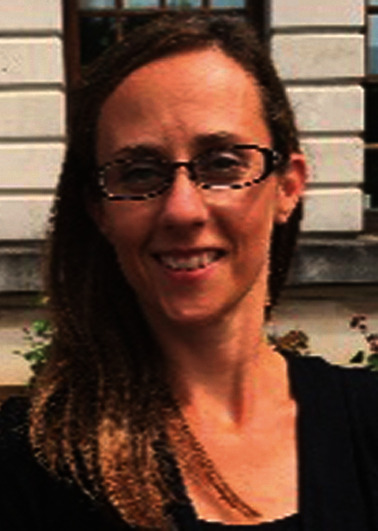

## Biographical Information

*Prof. Rebecca Melen completed her PhD degree at the University of Cambridge in 2012 with Prof. Wright. Following postdoctoral studies with Prof. Stephan in Toronto and with Prof. Gade in Heidelberg, she took up a position at Cardiff University in 2014. In 2018, she was awarded an EPSRC early career fellowship and she is the recipient of the 2019 RSC Harrison Meldola Memorial Prize and a 2022 Philip Leverhulme Prize in Chemistry. Her research interests lie in main group chemistry and catalysis*.

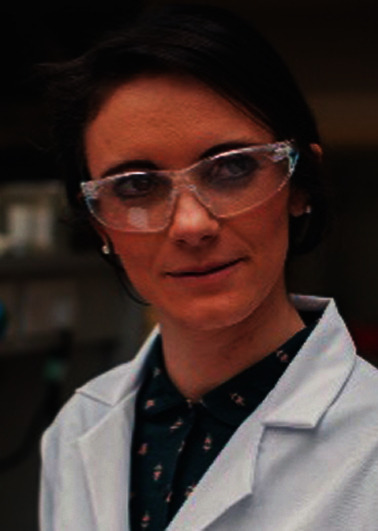

## Data Availability

Data sharing is not applicable to this article as no new data were created or analyzed in this study.
